# Developmental toxicant exposures and sex-specific effects on epigenetic programming and cardiovascular health across generations

**DOI:** 10.1093/eep/dvac017

**Published:** 2022-10-03

**Authors:** Laurie K Svoboda, Tomoko Ishikawa, Dana C Dolinoy

**Affiliations:** Environmental Health Sciences, University of Michigan, School of Public Health, 1415 Washington Heights, Ann Arbor, MI 48109, USA; Environmental Health Sciences, University of Michigan, School of Public Health, 1415 Washington Heights, Ann Arbor, MI 48109, USA; Environmental Health Sciences, University of Michigan, School of Public Health, 1415 Washington Heights, Ann Arbor, MI 48109, USA; Nutritional Sciences, University of Michigan, School of Public Health, 1415 Washington Heights, Ann Arbor, MI 48109, USA

**Keywords:** sex differences, toxicoepigenetics, DOHaD, cardiovascular disease, epigenetics

## Abstract

Despite substantial strides in diagnosis and treatment, cardiovascular diseases (CVDs) continue to represent the leading cause of death in the USA and around the world, resulting in significant morbidity and loss of productive years of life. It is increasingly evident that environmental exposures during early development can influence CVD risk across the life course. CVDs exhibit marked sexual dimorphism, but how sex interacts with environmental exposures to affect cardiovascular health is a critical and understudied area of environmental health. Emerging evidence suggests that developmental exposures may have multi- and transgenerational effects on cardiovascular health, with potential sex differences; however, further research in this important area is urgently needed. Lead (Pb), phthalate plasticizers, and perfluoroalkyl substances (PFAS) are ubiquitous environmental contaminants with numerous adverse human health effects. Notably, recent evidence suggests that developmental exposure to each of these toxicants has sex-specific effects on cardiovascular outcomes, but the underlying mechanisms, and their effects on future generations, require further investigation. This review article will highlight the role for the developmental environment in influencing cardiovascular health across generations, with a particular emphasis on sex differences and epigenetic mechanisms. In particular, we will focus on the current evidence for adverse multi and transgenerational effects of developmental exposures to Pb, phthalates, and PFAS and highlight areas where further research is needed.

## Introduction

### Developmental environment and cardiovascular disease

The developmental origins of health and disease (DOHaD) hypothesis posits that environmental insults during critical windows of development can lead to diseases later in life [1, 2]. While the majority of DOHaD studies have focused on pregnancy and lactation, development continues throughout childhood and puberty. Thus, vulnerability to environmental exposures under the DOHaD paradigm spans well beyond pregnancy. Likewise, a role for paternal environmental exposures in the programming of offspring health is increasingly evident but remains less studied [3, 4]. Early environmental insults have been linked to an increased risk of several non-communicable diseases later in life, including diabetes, cancer, neurodegenerative diseases, and the focus of this review—cardiovascular diseases (CVDs) [2, 5-7]. In this review article, we focus on CVDs, which refer to a broad array of health conditions, including atherosclerosis, myocardial infarction, stroke, cardiac arrhythmia, hypertension, heart failure, heart valve disease, and congenital heart defects [2, 8]. In many cases, as are noted throughout the manuscript, direct links between various chemicals and CVDs may not yet be established. However, we have noted that some are linked to conditions that are not classically considered CVD but are nevertheless intimately linked to cardiovascular health, such as obesity, disruptions in hormone signaling, and hyperglycemia [2, 8, 9]. For this reason, we have chosen to include these in the manuscript where relevant. It is important to note that, because CVDs are multifactorial, it is often difficult to ascertain whether effects of chemical exposures on cardiovascular health are direct or are secondary to effects on body weight, endocrine function, inflammation, and other factors. In vitro studies using traditional and new approach methodologies will likely shed light on this important distinction but are beyond the scope of this review.

The developing cardiovascular system is exquisitely sensitive to exogenous influences. Several decades ago, Dr David Barker observed that adverse conditions in utero increased the risk of coronary heart disease later in life, highlighting the importance of this critical stage of life in influencing long-term health trajectory. One of the most famous examples of Dr Barker’s work illustrating how early developmental environment affects cardiovascular health is the Dutch hunger winter of 1944–45, during which there was a restriction of food to the western part of the Netherlands as a result of World War II [10]. Women who were pregnant during this period were thus exposed to severe caloric restriction. Because medical records and food rations were well-documented, the effects of the famine on children born to women during this time have been extensively investigated. The Dutch Famine birth cohort documented that nutrient restriction *in utero* in the first trimester was associated with increased risk of obesity in women and dyslipidemia and cardiovascular disease in both sexes. Restriction in the third trimester, on the other hand, was associated with decreased risk of obesity in men, independent of birth weight [10-12]. Other studies in animal models have shown consistent effects of *in utero* nutrient restriction on epigenetic programming of genes controlling metabolic homeostasis and CVD risk [13]. Subsequent research has demonstrated that maternal obesity, hyperglycemia, and poor diet (high fat, high sucrose, or low protein) influence CVD risk in offspring [14-17]. Likewise, adverse childhood experiences have also been linked to poor cardiovascular health later in life [18-20]. In addition to nutritional and psychosocial factors, early exposure to environmental toxicants can also influence cardiovascular development and disease risk across the life course. A number of toxicants have been investigated in this regard, including air pollution [21], metals [22-24], phthalates [25, 26], perfluoroalkyl substances (PFAS) [27], pesticides [28], and hormone mimics such as diethylstilbestrol (DES) [29]. A summary of the factors discussed here can be found in Table 1.

**Table 1:** Developmental environmental factors linked to CVD

Environmental factor	Window of exposure	Model	Phenotype	Sex specificity	Reference
Famine	First trimester in utero	Human	Obesity, dyslipidemia (high LDL: HDL cholesterol ratio), cardiovascular disease (angina pectoris, Q waves on the ECG, or history of coronary revascularization)	Effect on obesity was observed in women at 50 years of age	[10,12]
Famine	Third trimester in utero	Human	Reduced risk of obesity	Study was conducted on men at 19 years of age	[11]
Obesogenic diet (high fat and high sugar) and obesity during pregnancy	Gestation and lactation	Mouse	Reduction in cardiac function at 32 weeks of age—hypertrophy and diastolic dysfunction	Only effects on male offspring were reported	[14]
Hyperglycemia	Gestation	Mouse	In embryos, impaired formation of left-right axis in heart and left-right asymmetry in developing organs	Not reported	[15]
High-fat and high-sucrose diet	Gestation and lactation	Mouse	Higher left ventricular weight in males and females, smaller left ventricular relative wall thickness in females, higher left ventricular relative wall thickness in males, cardiac mitochondrial abnormalities in both sexes	Yes	[16]
Protein restriction	Gestation and lactation	Rat	Decreased mitochondrial oxidative phosphorylation, decreased antioxidant capacity, and increased reactive oxygen species	Only male offspring were evaluated	[17]
Adverse childhood experiences (abuse, neglect, violence, and household dysfunction)	First 18 years of life	Human	Impaired endothelial function and reduced circulating levels of SIRT1	Only females were evaluated	[18]
Adverse childhood experiences (abuse, neglect, and household dysfunction)	First 18 years of life	Human	Increased risk of ischemic heart disease (mediated by psychosocial factors)	Sex differences not reported	[19]
Adverse childhood experiences (verbal and physical abuse, substance abuse, and poorly managed household)	First 18 years of life	Human	Increased multisystem health risk (high blood pressure, low heart rate variability, dyslipidemia, waist circumference, and other factors)	Sex differences not reported	[20]
PM_2.5_ exposure (73.61 μg/m^3^ for 6 h/day, 7 days/week)	Gestation	Mouse	Left ventricular remodeling and dysfunction, inflammation, fibrosis, altered calcium homeostasis at 3 months of age	Effects only observed in male mice	[21]
Pb exposure (0.2% in maternal drinking water)	Post-natal days 1–21 (birth until weaning)	Rat	Increased total cholesterol, LDL and triglycerides at 4 and 18 months of age; necrosis, immune cell infiltration at 4 and 18 months of age	Only male offspring were evaluated	[22]
Pb exposure	Measured in prenatal maternal toenail samples	Human	Increased risk of elevated systolic blood pressure in young children (average 5.5 years of age)	Effects were stronger among boys	[23]
Cadmium exposure (0, 1, and 50 ppm)	Gestation	Mouse	Increased relative heart weight at birth in both sexes, increased risk of hypertension in females at 6 months of age	Yes	[24]
DEHP (250 mg/kg, 500 mg/kg, and 1 g/kg)	Embryonic day E6.5 to E14.5	Mouse	Increased cardiac malformations (septal defects, ventricular myocardium noncompaction, and cardiac hypoplasia) at E15.5—potentially secondary to maternal toxicity and weight loss	Not reported	[25]
Phthalates and alkylphenolic compounds	Maternal occupational exposure assessed via job exposure matrix	Human	Increased incidence of congenital heart defects, particularly in children who also carried *ABCB1* polymorphism	Not reported	[26]
PFAS (PFOA and PFHxS)	Gestation (maternal serum and cord blood)	Human	Increased cardiometabolic risk score (insulin, waist circumference, and blood pressure) in adolescence	No sex differences were detected	[27]
DDT	Gestation (maternal serum samples)	Human	DDT exposure associated with hypertension at 39–47 years of age	Only females were evaluated	[28]
Diethylstilbestrol	Gestation	Human	Increased incidence of coronary artery disease and myocardial infarction	Only females were evaluated	[29]

### Sex differences in environment-induced cardiovascular disease

Although the early developmental environment plays a role in CVD risk, the effects of many environmental influences differ based on sex in both animals and humans [30-35]. However, the sex-specific effects of the developmental environment on cardiac health have received little attention. It is well-established that the incidence, pathogenesis, and prognosis of CVDs differ substantially between sexes [36, 37]. For example, although acute myocardial infarction is one of the leading causes of death in both men and women, women experience this condition later in life and exhibit atypical symptoms, resulting in delayed diagnosis [36]. Likewise, while ischemic heart disease is frequently characterized by coronary artery occlusion in men, women are more likely to exhibit microvascular dysfunction [37]. Hypertension is more common among men in young adults, but this disparity shifts in advanced age, where a greater number of women experience hypertension [38, 39]. Other conditions such as heart failure, genetic cardiomyopathies, and drug-induced arrhythmias also exhibit sexual dimorphism [37, 40, 41]. It is therefore imperative that we understand the sex differential effects that the early environment has on CVD risk and pathogenesis. Many factors noted above, including maternal obesity, diet, and diabetes [16, 42-44], psychosocial stressors [45], and toxicant exposures [46-48], show sex-specific effects on cardiovascular health. In this review, we will discuss what is known regarding the sex-specific multi and transgenerational effects of toxicant exposures on cardiac health. Each section contains a summary table of the studies discussed, which includes information on sex differences, if any, observed in each study.

### Epigenetics and multi and transgenerational inheritance

Epigenetics refers to the study of mitotically heritable changes in gene regulation that do not involve an alteration to the DNA sequence itself. Several mechanisms of epigenetic regulation have been identified, including modifications to the 5-position of cytosine bases in DNA (5-methylcytosine, 5-hydroxymethylcytosine, 5-carboxylcytosine, and 5-formylcytosine), modifications to histone proteins (methylation, acetylation, phosphorylation, and ubiquitination), and actions of non-coding RNA. The most extensively studied epigenetic modification is methylation of the 5-position of cytosine bases [5-methylcytosine (5mC)], which plays a role in X-inactivation, genomic imprinting, and repression of transposable elements [49-52]. The regulation of gene expression by DNA methylation is associated with genomic location. For example, DNA methylation at promoters is generally associated with repression of genes, while intragenic DNA methylation is associated with gene activation [53, 54]. The 5-hydroxymethylcytosine (5hmC) modification plays a critical role post-fertilization and in primordial germ cells (PGCs) during the dynamic reprogramming of DNA methylation [55-57] but has also been shown to be a stable epigenetic mark present in a variety of mammalian tissues [58-60], and we have shown that it is stably reprogrammed by perinatal exposures in mice and humans [61, 62].

**Figure 1:**
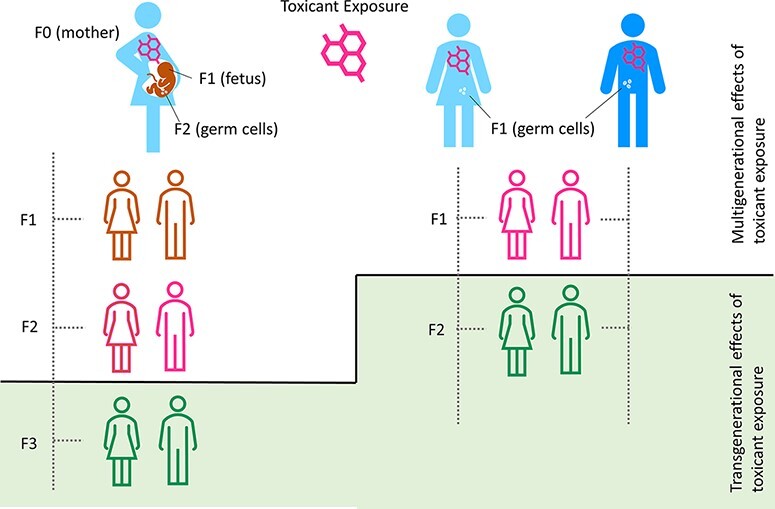
multigenerational vs. transgenerational effects. Left: In a pregnant (F0) individual exposed to a toxicant, the F1 and F2 generations will also receive direct toxicant exposure, resulting in multigenerational effects. Transgenerational effects are those that occur in generations not directly exposed to the toxicant, i.e. F3 and beyond. Right: In males and non-gestating females, the F0 individual and F1 offspring are directly exposed (via germ cells), and transgenerational effects are observed in the F2 generation and beyond. The sex-specific effects of toxicants on transgenerational epigenetic inheritance are poorly understood

Two distinct waves of epigenetic reprogramming occur during early development, making this period vulnerable to environmental perturbations [63, 64]. The first wave of reprogramming occurs post-fertilization, in which parental epigenetic marks are erased and the somatic epigenetic marks of the developing embryo are established [65, 66]. The second wave of reprogramming occurs in the fetal primordial germ cells, in which sex and parent of origin-specific epigenetic patterning are established [67]. Environmental disruptions in the normal epigenetic patterning in germ cells that are not corrected during the development of the future generation may result in multi and transgenerational effects [68]. In a pregnant (F0) individual, toxicant-induced epigenetic changes may occur in their somatic and germ cells, as well as in the somatic and primordial germ cells of the developing fetus (Fig. 1) [68]. Toxicant exposure-induced effects occurring in the offspring (F1) and grand offspring (F2) generations are termed multigenerational effects, as F0 and F1 generations receive direct exposure during the pregnancy, and the primordial germ cells of the F2 generation are also directly exposed (Fig. 1) [68]. In contrast, transgenerational epigenetic inheritance refers to the transmission of environmental effects to future generations that did not receive the exposure directly through embryonic development or germ cells, i.e. F3 generations and beyond (Fig. 1) [68]. In males and non-gestating females, the F0 individual and F1 offspring are directly exposed, and transgenerational effects are observed in the F2 generation and beyond (Fig. 1). Transgenerational effects of environmental exposures have been reported in nematodes, plants, fruit flies, and mammals [68-71]. However, the prevalence of this phenomenon and its relevance to health, in particular human health, is the subject of significant debate and ongoing study [72, 73].

**Figure 2:**
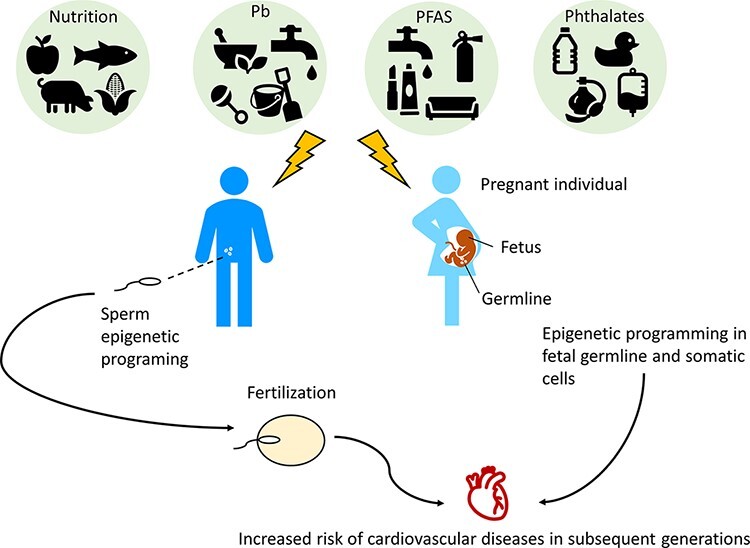
Diet and exposure to toxicants such as Pb, PFAS, and phthalates can affect the epigenome of the parental germ cells, as well as the somatic and germline cells of a developing fetus, resulting in multigenerational and transgenerational effects on cardiovascular disease risk. The sex-specific generational effects of Pb, PFAS, and phthalates have not been adequately investigated

### Effects of environment on cardiovascular health across generations

Several animal and human studies provide evidence that ancestral environmental exposures may induce cardiovascular health effects that span generations (Fig. 2). A summary of the manuscripts reviewed in this section can be found in Table 2. In animal studies, F1, F2, and F3 male progeny of rats exposed to global nutrient and calorie restriction during their entire pregnancy exhibited cardiovascular dysfunction, including high blood pressure, altered nitric oxide production, and impaired vasodilation in response to acetylcholine [74]. Maternal obesity may also produce transgenerational effects on cardiac health. Obese female mice produced both male and female offspring from F1 to F3 with cardiac defects, including mitochondrial dysfunction and increased left ventricular mass [16]. Epigenetic or other mechanisms were not investigated in either of these studies, however, so the underlying molecular basis for their observations remains unclear. In zebrafish, paternal exposure to bisphenol A resulted in cardiac defects (pericardial edema, malformations in both cardiac chambers, ectopic heartbeats, and arrhythmias) in F1 progeny as well as altered expression of cardiac developmental genes [75]. Cardiac edema was also observed in F2 progeny exposed to the highest dose of BPA [75]. The authors observed no significant changes in global DNA methylation in sperm and testicular tissue from control vs. treated animals; however, other epigenetic or site-specific DNA methylation changes were not investigated [75].

**Table 2:** Generational effects of environment on cardiovascular health

Environmental factor	Window of exposure	Model	Epigenetic/molecular effect	Phenotype	Sex specificity	Reference
Nutrient and calorie restriction	Gestation	Rat	Not investigated	High blood pressure and impaired vasodilation (F1, F2, and F3) at 16 weeks of age	Only male offspring were evaluated	[74]
High-fat and high-sucrose diet	Gestation and lactation	Mouse	Not investigated	Increased left ventricular mass in male (F1, F2, and F3) and female (F1 and F2) offspring, cardiac mitochondrial abnormalities in both sexes (F1, F2, and F3) at 8 weeks of age	Yes	[16]
Bisphenol A (100 and 2000 μg/l)	Spermatogenesis	Zebrafish	No changes in global DNA methylation in testicular cells or spermatozoa	Cardiac edema and malformations (defects in both heart chambers) in high dose exposed 7 dpf larvae (F1 and F2)	Not reported	[75]
Food availability	Slow growth period (before pre-pubertal peak in growth velocity) in males	Human	Not investigated	Increased risk of death from diabetes mellitus in grandchildren of men exposed to abundant food during the slow growth period; decreased risk of death from CVD among grandchildren of men exposed to famine during the slow growth period	Not reported	[76]
Food availability	Period prior to puberty in females	Human	Not investigated	Increased risk of cardiovascular mortality in daughters of the sons from women exposed to sharp changes in food supply before puberty	Yes	[77]
Famine	Gestation	Human	Not investigated	Increased risk of hyperglycemia (F1 and F2) in adulthood	Not reported	[78]
BPA, DEHP, and DBP at two doses: BPA 50 mg/kg BW/day, DEHP 750 mg/kg BW/day and DBP 66 mg/kg/BW/day and a second group with half of those doses via intraperitoneal injection	Gestation days 8–14	Rat	Differential DNA methylation in 197 regions in sperm of F3 generation	Obesity and pubertal abnormalities (primarily delayed puberty onset) in F3 males and females	Effects observed in both males and females	[82]
Methoxychlor (200 mg/kg BW/day)	Gestation days 8–14	Rat	Differential DNA methylation in sperm of F3 generation	Pubertal abnormalities in F1 males and females; obesity in F3 males and females, with a greater incidence in males; obesity in F4 generation males (transmitted through female)	Yes	[83]
A pesticide mixture (permethrin 150 mg/kg and DEET 40 mg/kg), a plastic mixture (bisphenol A 50 mg/kg, DBP 66 mg/kg, and DEHP 750 mg/kg), dioxin (TCDD 100 ng/kg) and a hydrocarbon mixture (jet fuel, JP8 500 mg/kg) or sesame oil control by intraperitoneal injection	Gestation days 8 to 14	Rat	Differentially methylated regions in the sperm of males from all exposure lineages	Early onset puberty in F3 females with exposure to plastics, dioxin and jet fuel; reduced number of ovarian follicles in F3 females with all exposures; increased sperm apoptosis in F3 males exposed to jet fuel	Yes	[84]
Vinclozolin (100 or 200 mg/kg) or DMSO in sesame oil control	Gestation days 7–13	Mouse	Differential DNA methylation in F3 sperm	Increased testis, prostate and kidney disease in F3 males; increase sperm apoptosis in F1, F2, and F3 males; increased ovarian cysts in F1, F2, and F3 females	Yes	[85]
Jet fuel (25% the oral LD50 dose) or DMSO in sesame oil by intraperitoneal injection	Gestation days 8 to 14	Rat	Differential DNA methylation at 33 regions in F3 sperm	Renal abnormalities in F1 males and females; prostate and altered puberty timing in F1 males; loss of ovarian follicles and polycystic ovarian disease in F1 and F3 females; obesity in F3 males and females	Yes	[86]
Glyphosate (25 mg/kg body weight daily)	Gestation days 8–14	Rat	Differential DNA methylation in sperm of F1 (264 regions), F2 (174 regions), and F3 (378 regions) generations	Delayed puberty in F1 and F2 males and F2 females; obesity in F2 and F3 males and females at 1 year of age	Yes	[87]
Tributyltin (50 nM in drinking water)	Gestation and lactation	Mouse	Changes in global DNA methylation in white adipose tissue from F4 males; changes in chromatin accessibility in F3 and F4 sperm	Increased obesity in response to a high-fat diet in F4 males	Yes	[6]
Tributyltin (5.42, 54.2, or 542 nM in drinking water)	7 days prior to mating, through gestation	Mouse	Not investigated	Increased white adipose tissue and depots and adipocyte size and number in F1, F2, and F3 males and F1 and F2 females	Effects observed in both males and females	[88]
Maternal high-fat diet	6 weeks prior to mating, through gestation and lactation	Mouse	Not investigated	Increased body weight in F3 females and improved glucose tolerance in F3 males	Yes	[89]

Human studies provide further support for the hypothesis that the cardiovascular effects of the environment can span multiple generations. Research conducted on three cohorts of people born in 1890, 1905, and 1920 in Overkalix, a Municipality in Sweden, revealed that a dearth of food during a father’s slow growth period (the period between ages 9 and 12 years) resulted in a reduced risk of mortality from cardiovascular disease in the children [76]. However, if the paternal grandfather was exposed to an abundance of food during this critical period, then his grandchildren had a higher risk of mortality from diabetes [76]. Further investigation of this cohort of people showed that if a paternal grandmother lived through a period of sharp changes in food availability in early life up to puberty, then the daughters of her sons exhibited an increased risk for cardiovascular mortality [77]. In a separate study of a population exposed to the Chinese Famine (1959–61), prenatal exposure to famine was associated with an increased risk of hyperglycemia in adulthood for the exposed, as well as their offspring [78]. The effects on offspring hyperglycemia risk were independent of sex and occurred in offspring of both exposed mothers and fathers [78]. The molecular mechanisms underlying these effects are unclear; however, studies have demonstrated that epigenetic factors such as DNA methylation may underlie the transmission of stress across generations [79, 80].

Multi- and transgenerational effects of toxicant exposures have been reported for a wide variety of chemicals, including BPA [81, 82], phthalates [82], parabens, pesticides [83] and dioxin [84], fungicides [85], and jet fuel [86]. The transgenerational effects of chemical exposures on long-term cardiovascular health are poorly understood, and given the significant burden of morbidity and mortality posed by CVDs, further investigation into this important topic is urgently needed. Although a relatively small number of studies have investigated the transgenerational effects of environment specifically on cardiac health, significant animal and human evidence also links ancestral environment to conditions closely associated with CVD, including obesity, diabetes, and reproductive dysfunction. For example, ancestral exposure to BPA and phthalates [82], pesticides/herbicides [83, 87], tributyltin [6, 88], and high-fat diet [89] have been linked to an increased risk of obesity or adiposity, and some of these exposures exhibited sex differences. Obesity induced by methoxychlor was present in both F3 males and females, with a greater incidence in males [83]. Offspring consumption of a high-fat diet led to obesity in male but not female F4 mice after ancestral exposure to tributyltin [6]. A maternal high-fat diet led to increased body weight in F3 female mice, which was transmitted through the paternal line [89]. On the other hand, glyphosate and plastics-induced obesity was present in both F3 males and females at roughly equal frequency [82, 87]. These studies identified chemical-induced epigenetic changes that were present in the generations that did not receive direct exposure, providing evidence that transgenerational inheritance may be mediated by changes to the germline epigenome. Indeed, methoxychlor and BPA/phthalate-induced phenotypes were accompanied by changes in DNA methylation in the sperm of F3 mice [83, 84]. Glyphosate exposure led to altered DNA methylation in F1–F3 sperm [87]. Effects of ancestral exposure to tributyltin were associated with changes in DNA methylation and chromatin accessibility in F3 and F4 sperm and adipose tissue [6]. Increased body weight as a result of maternal high-fat diet was accompanied by changes in expression of paternally expressed imprinted genes, suggesting that stable epigenetic modifications at these genes may have been responsible for the observed effects on gene expression and body weight [89].

In the following sections, we will describe the sex-specific effects of developmental exposure to Pb, PFAS, and phthalates on cardiovascular health and epigenetic programming in human and animal studies, as well as the current evidence available regarding the transgenerational effects of these chemicals on cardiovascular health.

### Developmental Pb exposure and cardiovascular health

The metal Pb is a ubiquitous environmental contaminant with a large number of deleterious human health effects. Sources of human Pb exposure include soil, water, food, contaminated household dust, consumer goods, folk remedies, smoking, and industrial sources [90, 91]. Human exposure to Pb occurs primarily through ingestion or inhalation, where it causes adverse neurological, hematological, renal, and cardiovascular effects [91]. A summary of the cardiovascular and epigenetic effects of developmental Pb exposure can be found in Tables 3 and 4, respectively.

**Table 3:** Developmental Pb exposure and cardiovascular health

Exposure details	Window of exposure	Model	Epigenetic/molecular effect	Phenotype	Sex specificity	Reference
0.2% Pb acetate in drinking water	Gestation until adulthood (12, 20, and 28 weeks)	Rat	Not investigated	Elevated blood pressure, respiratory frequency, hyperactive baro- and chemoreflexes in adulthood in both sexes	Effects observed in both males and females	[96]
500 ppm of Pb acetate in drinking water	Gestation and lactation	Rat	Not investigated	Elevated systolic blood pressure, increased reactivity to noradrenaline in adolescent and adult male offspring	Only male offspring were evaluated	[97]
0.05% Pb acetate in drinking water	1 week prior to mating until day 11.5 gestation	Mouse	Not investigated	Abnormal angiogenesis in yolk sac of embryos	Not investigated	[98]
2.1 (low), 16 (medium), or 32 (high) ppm Pb acetate in drinking water	2 weeks prior to mating through gestation and lactation	Mouse	Not investigated	Increased food intake and energy expenditure in both sexes in adulthood, increased body weight, body fat, and insulin response in males	Yes	[100]
12 mg of Pb acetate per ml or sodium acetate control in drinking water	30 days prior to breeding until 21 days of age	Rat	Not investigated	Delayed timing of puberty	Only female offspring were evaluated	[101]
300 mg/l Pb acetate in drinking water	Gestation and lactation	Rat	Not investigated	Elevated blood glucose at 12 and 21 days post-natal	Not reported	[102]
100 mg Pb/kg diet	8 weeks treatment starting at 35 days of age	Rabbit	Not investigated	Reduced relative heart weight, increased creatine phosphokinase	Only males were evaluated	[103]
1% Pb acetate in drinking water	12 and 40 day treatments starting at 4-5 weeks of age	Rat	Increased levels of acetylated histone H3 in abdominal and thoracic aortas and cardiac tissue	Increased proliferating cell nuclear antigen in cardiac tissue, enlarged cardiac cells after 40 days, elevated blood pressure during first 17 days, altered internal elastic lamina of aorta	Not reported	[104]
60 ppm of Pb acetate in drinking water	Weaning until 10 months of age	Rat	Not investigated	Increased diastolic and systolic blood pressure, increased cardiac inotropy	Only males were evaluated	[105]
Blood Pb levels in preschool	Early childhood	Human	Not investigated	Smaller left ventricle (interventricular septum, left ventricular posterior wall, and left ventricular mass index); impaired left ventricular systolic function (fractional shortening and ejection fraction); increased inflammatory cell counts in both sexes	Effects observed in both males and females	[109]
Maternal hair Pb levels	Gestation	Human	Not investigated	Increased risk of congenital heart defects (septal, conotruncal, right-sided obstructive, left-sided obstructive, anomalous venous return, and others)	Not reported	[110]
Parental hair, toenail, and tooth Pb levels	Gestation	Human	Not investigated	Increased Pb levels in parents of children with congenital heart defects	Not reported	[111]
Blood Pb levels in pregnant women at 17–40 weeks gestation	Gestation	Human	Not investigated	Increased blood Pb levels in mothers of children with congenital heart defects (conotruncal defects, right ventricle outflow tract obstructions, and septal defects)	Not reported	[112]
Pb levels in first meconium samples of newborns	Gestation	Human	Not investigated	Increased Pb levels in meconium of infants with conotruncal heart defects	Not reported	[113]
Pb levels in blood of mothers with infants under 6 months of age	Gestation	Human	Not investigated	Increased blood Pb levels in mothers of infants with congenital heart defects	Effects observed in both males and females	[114]
Pb levels in prenatal cord blood and early childhood	Gestation and early childhood	Human	Not investigated	Elevated cord blood Pb associated with increased systolic blood pressure; elevated early childhood Pb associated with increased peripheral vascular resistance and decreased stroke volume in response to acute stress	Not reported	[115]
Blood Pb levels in childhood (9–11 years of age)	Early childhood	Human	Not investigated	Impaired response to cardiovascular stress (reduced stroke volume and increased peripheral vascular resistance)	Not reported	[116]
Pb levels in maternal toenail samples collected at ∼28 weeks gestation and/or 6 weeks postpartum	Early and late prenatal periods	Human	Not investigated	Early prenatal Pb exposure associated with significantly higher systolic blood pressure in children (mean age 5.5 years), with stronger effects among boys compared to girls	Yes	[47]
Blood Pb levels in pregnant women in second trimester	Gestation	Human	Not investigated	Higher maternal blood Pb associated with higher risk of elevated systolic blood pressure in children 4–6 years of age who had short gestation	Not reported	[117]
Pb in maternal urine at ∼12 weeks gestation	Gestation	Human	Not investigated	Increased risk of elevated blood pressure in early childhood (4–11 years of age), particularly in combination with molybdenum	Not reported	[118]
Maternal blood Pb concentrations at gestational weeks 14 and 30	Gestation	Human	Not investigated	Late gestation Pb associated with reduced kidney volume in children ∼4.5 years of age, with a stronger association in girls	Yes	[120]
Maternal blood Pb levels	Gestation	Human	Not investigated	Lower *Z*-scores for total cholesterol, LDL and HDL in boys with maternal blood Pb levels ≥ 5 µg/dl	Yes—No effects noted in girls	[121]

**Table 4:** Developmental Pb exposure and the epigenome in the context of cardiovascular health

Exposure details	Window of exposure	Model	Epigenetic/molecular effect	Phenotype	Sex specificity	Reference
Pb concentrations in maternal urine at gestational week 8 and erythrocytes at gestational week 14	Early gestation	Human	Significant inverse association between urinary Pb and cord blood DNA methylation at nine CpGs, including several in *Glycoprotein 6* gene	Not investigated	Effects observed in both sexes	[129]
Pb levels in prenatal maternal red blood cells (average 27.9 weeks gestation) and cord blood	Mid-late gestation	Human	Significant negative association between maternal RBC Pb and cord blood DNA methylation at four CpGs in both sexes; several differentially methylated CpGs specific to males and females, including *NOTCH1* and *GNAS* in females	Not investigated	Yes—More significantly differentially methylated CpGs in female vs. male infants	[132]
Maternal blood Pb at ∼12 weeks gestation	Early gestation	Human	Hypermethylation of the *MEG3* imprinted regulatory domain in umbilical cord blood	Lower birth weight and rapid gains in adiposity at 2–3 years of age	Not reported	[133]
Serial blood Pb samples from birth to 78 months of age	Early childhood	Human	Mean blood Pb levels in childhood associated with a significant decrease in DNA methylation at *IGF2/H19* and *PEG3* loci and increased methylation at *PLAGL1/HYMAI* in peripheral blood in adulthood	Yes—Effects on *PEG3* stronger in males; effects on *IGF2/H19* stronger in females	Yes	[136]
0, 100, or 500 ppb of Pb in petri dish water from <1 to 2 until 72 h post-fertilization (end of embryogenesis)	Embryogenesis	Zebrafish	Significant reduction in global DNA methylation in whole embryos at both doses; reduced transcript expression of *dnmt3* and *dnmt4* (human *DNMT3B* orthologs)	Not investigated	Not reported	[138]
0, 2, 10, and 17 μg/l of Pb(NO_3_)_2_ in culture plate	3–4 h to 7 days post-fertilization	Zebrafish	Down-regulation of *Dnmt1* and *Dnmt3b* transcript expression in embryos	Increased malformations and decreased heart rate with Pb in combination with elevated temperature	Not reported	[139]
Pb acetate in drinking water	2 weeks prior to mating, through gestation and lactation	Mouse	Sex-specific differential methylation of hundreds of CpGs and 1000 base pair regions in male and female offspring hearts in adulthood	Not investigated	Yes	[146]

#### Cardiovascular effects of Pb exposure in animals

Pb exposure in animals outside of the developmental period is widely reported to cause cardiotoxicity, heart failure, and hypertension [92-95]. Developmental exposures have been less extensively studied, but several have demonstrated adverse effects on blood pressure, cardiac function, and metabolic parameters. First, developmental Pb exposure in rats leads to hypertension in both sexes [96] or in male offspring (females were not included in the study) [97]. Exposure to Pb is also associated with altered angiogenesis in mouse embryos, suggesting that some Pb-induced developmental defects may be mediated by impaired blood flow [98]. Pb exposure during lactation impaired cardiac mitochondrial function and depleted antioxidant capacity in male rats [22], consistent with data in humans demonstrating direct effects of Pb on cardiac function [99]. Several additional studies have demonstrated that Pb exposure causes sex-specific changes in metabolic parameters that are closely linked to cardiovascular health, including weight, food intake, insulin signaling, blood glucose levels, and circulating IGF-1 [100-102]. Several additional Pb exposure studies have been conducted in young, recently weaned animals, a developmental period in which significant growth and hormonal changes are still occurring. Newly weaned male rabbits treated with Pb for 8 weeks showed serum and histological evidence of cardiac injury [103]. Young male rats exposed to Pb exhibited pathological changes in the structure of the aorta and myocardium [104]. Chronic Pb exposure in young male mice led to increased cardiac inotropy and blood pressure, concomitant with changes in circulating enzymes critical for blood pressure regulation [105].

#### Cardiovascular effects of Pb exposure in humans

Although investigations into the effects of Pb on health have focused primarily on neurological outcomes, numerous studies also link Pb exposure to adverse cardiovascular outcomes, including high blood pressure, myocardial infarction, coronary artery disease, cardiac arrhythmias, heart failure, atherosclerosis, and stroke [99, 106-108]. Developmental exposures to Pb have also been linked to cardiovascular diseases in humans. First, there is evidence that Pb alters normal cardiac development. Children exposed to Pb in e-waste had smaller left ventricles, decreased left ventricular function, and increased markers of inflammation, effects that were independent of sex [109]. Maternal Pb exposure has also been associated with congenital heart defects in several studies, although sex differences were not investigated [110-113], or effects did not differ by sex [114]. Cardiovascular function and reactivity are tightly regulated by the autonomic nervous system, and several lines of evidence link Pb exposure to altered autonomic regulation. Blood Pb levels were associated with significant autonomic and cardiovascular dysregulation in response to acute psychological stress in children, with some sex differences [115, 116]. Like adults, studies have also demonstrated that Pb exposure may lead to increased blood pressure in children. In utero exposure to Pb is associated with increased baseline blood pressure in children [47, 117, 118], with sex-dependent [47] and independent [117] associations observed. Importantly, some reported effects of Pb were significant at Pb levels well below 10 μg/dl, levels that are still prevalent in the USA, particularly among urban minority populations [47, 117, 119]. Cardiovascular and renal functions are intimately linked, and Pb exposure was linked to altered kidney volume in children, particularly in females [120]. Finally, Pb may also impact cardiac health through alterations in blood lipids, an effect observed only in male children [121]. Although developmental Pb exposure has been linked to neurological defects later in life [5], the effects of early Pb exposure on adult cardiovascular health have not been investigated. Importantly, individuals in the USA who were highly exposed to Pb as children in the 1960s, 1970s, and early 1980s [122] are now in middle age, a stage of life when cardiovascular diseases emerge. It is thus imperative that the scientific community better understand the effects of early Pb exposure on long-term cardiovascular health.

#### Effects of developmental Pb exposure on the epigenome

Pb exposure throughout the life course has been linked to alterations in DNA methylation, histone modifications, and non-coding RNAs in various tissues [123]. Significant evidence also links developmental Pb exposure to epigenetic alterations in offspring. Human studies investigating the effects of developmental Pb exposure on the epigenome have focused primarily on the measurement of Pb in maternal blood, maternal tibia or patella, cord blood, or neonatal blood spots as proxies for Pb exposure during pregnancy. These studies have repeatedly demonstrated that Pb exposure is associated with changes in DNA methylation and hydroxymethylation in offspring blood [62, 124-126], and sex differences have been reported [125-127]. Moreover, recent work suggests that maternal Pb-induced changes in DNA methylation may be transmitted to grandchildren [128].

Few studies to date, however, have investigated the effects of Pb on the epigenome in the context of the cardiovascular system and cardiovascular disease. Pb-associated changes in DNA methylation have been identified at genes relevant to cardiovascular disease, although the long-term implications of these findings for cardiovascular health are unclear. For example, developmental Pb exposure leads to a sex-independent reduction in methylation of the glycoprotein VI gene, which plays an important role in platelet activation and blood clotting [129]. Likewise, maternal blood Pb exposure was associated with altered cord blood DNA methylation at numerous CpGs, particularly in female infants, including several associated with cardiovascular development and diseases such as *NOTCH1* and *GNAS* [130-132].

Developmental Pb exposure-induced changes in DNA methylation at imprinted genes, which play a critical role in growth and cardiometabolic health, have also been reported. In addition to changes in DNA methylation at the imprinted *GNAS* locus noted above [132], maternal blood Pb levels were associated with altered DNA methylation of the *MEG3* imprinted gene in cord blood, concomitant with reduced birth weight and rapid increases in adiposity, both of which are associated with long-term risk of cardiovascular disease [133]. Given the small sample size, sex differences were not identified. Roles for *MEG3* have been identified in the regulation of cardiac and vascular remodeling in the context of cardiac fibrosis and angiogenesis [134, 135]. Prenatal Pb exposure is also associated with changes in DNA methylation in the imprinted gene *IGF2* [136], which have also been implicated in cardiovascular disease [137].

Perinatal Pb exposure has also been linked to epigenetic changes in animal models, although effects on cardiovascular tissues have not been adequately investigated. Pb exposure affects Dnmt expression and activity in zebrafish embryos [138, 139]. In particular, Pb exposure and thermal stress cooperate to affect heart rate in zebrafish embryos, concomitant with altered expression of Dnmt1 and Dnmt3b [139]. Although these studies did not address cardiovascular disease directly, modulation of the functions of Dnmt1, Dnmt3a, and Dnmt3b has been linked to atherosclerosis and heart failure in animal models [140, 141], and single nucleotide polymorphisms in *DNMT1* are associated with coronary artery disease risk in humans [142]. In mice, Pb exposure during pregnancy and lactation led to sex and tissue-specific alterations in DNA methylation at murine IAP transposons, although heart tissue was not examined [143]. Given the established role for Pb as a neurotoxicant, multiple studies have demonstrated that perinatal Pb exposure causes epigenetic changes in rodent brain [144, 145]. Few studies, however, have investigated the effects of Pb on the cardiac epigenome. We recently discovered that perinatal Pb exposure results in sex-specific changes in DNA methylation in the hearts of offspring mice that are present in adulthood, long after cessation of the exposure [146]. The implications of these changes for cardiac health are under investigation.

### Developmental PFAS exposure and cardiovascular health

PFAS are a large family of manmade chemicals with a common structure, consisting of a chain of carbon atoms bound to fluorine atoms, with a functional group at the end of the molecule [147]. Because of the strength of the carbon fluorine bond, PFAS are environmentally persistent, with long half lives in the human body and in the environment [147]. PFAS are widely used in the design of consumer products that resist grease, water, stains, and oil, as well as firefighting foams and some cosmetics [147]. Humans are exposed to PFAS primarily through ingestion of food and water, inhalation of indoor air, and contact with other contaminated sources [147]. PFAS chain lengths vary, with longer chain chemicals exhibiting longer half lives in the human body [147]. The health effects of longer chain legacy PFAS such as perfluorooctanoic acid (PFOA) and perfluorooctane sulfonic acid (PFOS) are the most extensively studied, while the effects of shorter chain PFAS on human health are still poorly understood. A summary of the cardiovascular and epigenetic effects of developmental PFAS exposures can be found in Tables 5 and 6, respectively.

**Table 5:** Developmental PFAS exposure and cardiovascular health

Exposure details	Window of exposure	Model	Epigenetic/molecular effect	Phenotype	Sex specificity	Reference
PFOA at dose of 2 mg/kg egg weight injected into eggs	Embryonic days 0–10	Chicken	Not investigated	Altered cardiomyocyte size, increased intracellular calcium	Not reported	[148]
PFOA at doses of 0, 0.5, 1, and 2 mg/kg of egg weight injected into eggs	Embryonic days 0–19	Chicken	Not investigated	Thinning of the right ventricular wall in 1 and 2 mg/kg dose groups relative to vehicle control in 19-day embryo hearts; changes in ventricular wall thickness, increased left ventricular mass, and increased heart rate in 1-day-old hatchling hearts	Not reported	[149]
PFOA (2 mg/kg) or HFPO-DA (1, 2, 4, and 8 mg/kg) injected into eggs	Embryonic days 0–21	Chicken	Not investigated	Thinning of the right ventricular wall and elevated heart rates with PFOA or HFPO-DA treatment	Not reported	[150]
PFuDA, PFDA, PFNA, and PFOA in doses 10 μM to 2 mM	Embryonic days 0–10	Xenopus	Not investigated	Pericardial edema at embryonic stage 26, heart malformations (enlarged atrium, loss of atrial septum, and thinner atrial and ventricular walls) in animals exposed to 130 μM PFDA or PFuDA	Not reported	[151]
PFOS at doses of 0.1, 0.6, and 2.0 mg/kg/day by oral gavage	Gestational days 2–21	Rat	Not investigated	Dose-dependent increase in apoptosis in heart at post-natal day 21	Not reported	[152]
PFOA 1, 10, and 20 mg/kg via intraperitoneal injection	Gestational days 5–9	Mouse	Not investigated	Cardiac mitochondrial swelling and reactive oxygen species at day 15 gestation	Not reported	[153]
1, 25, 50, and 100 μg/l PFOA in culture medium	2–30 h post-fertilization	Zebrafish	Not investigated	Reduced heart rate at 48 h post-fertilization for 25, 50, and 100 μg/l PFOA; increased apoptosis in heart at all doses at 72 h post-fertilization	Not reported	[154]
HFPO-DA at 1–125 mg/kg/day or 10–250 mg/kg/day via oral gavage	Gestational days 16–20 for 1–125 mg/kg/day and gestational days 8 until post-natal day 2 for 10–250 mg/kg/day	Rat	Not investigated	Elevated offspring triglycerides and cholesterol at highest doses for gestational day 8 to post-natal day 2 exposure	Not reported	[155]
PFOA at 0, 0.01, 0.1, 0.3, 1, 3, or 5 mg PFOA/kg body weight via oral gavage	Gestational days 1–17	Mouse	Not investigated	Increase in body weights in low dose exposed females at 20–29 weeks of age; increased serum insulin and leptin at lower doses at 21–33 weeks of age	Only data from females were reported in this manuscript	[156]
Six PFAS (Me-PFOSA-AcOH, PFDA, PFNA, PFOS, PFOA, and PFHxS) in maternal serum during third trimester	Late gestation	Human	Not investigated	Positive association between several PFAS and total cholesterol and triglycerides in pregnant women	This study investigated pregnant women	[157]
Levels of 10 PFAS (PFOS, PFOA, PFNA, PFDA, PFUnDA, PFHxS, PFDoA, PFBS, PFHpA, and PFOSA) in maternal blood during early pregnancy	Early gestation	Human	Not investigated	Increased risk of gestational diabetes with PFBS, PFOS, and PFHpA; positive correlation between PFOS, PFNA, PFDA, PFHxS, and PFHpA and glucose levels in oral glucose tolerance test; PFAS mixtures associated with abnormal glucose homeostasis	This study investigated pregnant women	[158]
PFHxS, PFOS, and PFOA), PFNA, and PFDA in maternal serum	Early gestation	Human	Not investigated	PFAS exposures (except PFHxS) associated with higher systolic and diastolic blood pressures (not all associations significant)	This study investigated pregnant women	[159]
First trimester maternal plasma levels of PFOA, PFOS, and PFHxS	Early gestation	Human	Not investigated	Increased risk of preeclampsia with PFHxS in women carrying female fetuses; increased risk of hypertension with PFOS and PFHxS in women carrying male fetuses	This study investigated pregnant women; associations differed based on fetal sex	[160]
Levels of 27 PFAS in maternal blood and cord blood plasma before and during delivery	Late gestation	Human	Not investigated	Increased odds of septal defects with exposure to 6 m-PFOS or PFDA in maternal blood; increased odds of conotruncal defects with exposure to PFOS or PFDoA in maternal blood	Not reported	[165]
Estimated serum PFOA concentration during pregnancy	Gestation	Human	Not investigated	Weak association between maternal serum PFOA concentrations and congenital heart defects	Not reported	[166]
Estimated serum PFOA concentration during pregnancy	Gestation	Human	Not investigated	No significant association between maternal serum PFOA concentrations and congenital heart defects	Not reported	[167]
PFOA exposure, assessed by Little Hocking Water Association service category	Gestation	Human	Not investigated	No significant association between maternal PFOA exposure and congenital defects of any kind	Not reported	[168]
Serum levels of 12 PFAS in children between 12 and 20 years of age	Adolescence	Human	Not investigated	Strong positive association between PFOS and diastolic blood pressure in males in linear model, but no significant association in non-linear model	Yes—Effects in males but not females	[48]
Serum levels of PFOA, PFOS, PFHxS, and PFNA in obese children 8–12 years of age	Pre-adolescence	Human	Not investigated	Risk of elevated systolic blood pressure and LDL/total cholesterol with PFNA; elevated LDL cholesterol with PFOA and PFOS; elevated total cholesterol with PFOA	No	[169]
Serum concentrations of 18 PFAS in adolescents aged 15–19 years	Adolescence	Human	Not investigated	Positive association between PFOS, PFNA, PFDA, PFUnDA, and apolipoprotein B, total- and LDL cholesterol; positive association between total PFAS, PFOS, PFNA, PFDA, PFUnDA, and risk of dyslipidemia; positive association between total PFAS, PFHxS, PFOS, PFOA, and hypertension; positive association between PFHpS and PFHxS and risk of obesity	Not reported	[170]
Levels of PFHxS, PFOS, PFOA, and PFNA in first trimester maternal plasma	Early gestation	Human	Not investigated	Positive association between prenatal PFHxS and triglycerides; positive association between PFNA and cardiometabolic risk score at 4 years of age	No	[171]
Serum concentrations of 12 PFAS in adolescents aged 14–19 years or children aged 8–11 years	Childhood and adolescence	Human	Not investigated	Increased total and LDL cholesterol with all PFAS in adolescents; increased total, LDL and HDL, cholesterol with PFOS and PFNA in children; increased HDL cholesterol with PFOA and PFHxS in children; lower BMI z-score with higher concentrations of PFAS	Yes—Stronger associations observed in girls	[172]
Levels of 5 PFAS in cord blood in World Trade Center birth cohort	Gestation	Human	Not investigated	Higher total cord blood lipids with PFOS, PFOA, and PFHxS levels; higher total cholesterol and lower triglycerides with PFDS; higher triglycerides with PFOA and PFHxS exposure	Not reported	[173]
Blood PFOS, PFHxS, and PFOA levels in children 8–14 years of age	Childhood–adolescence	Human	Not investigated	Increased blood glucose (2 h oral glucose tolerance test) with PFOA and PFHxS, or increased glucose area under the curve for PFHxS; significant alterations in lipids and amino acids with PFAS exposure	No	[174]
PFAS levels in maternal plasma (median 9.7 weeks gestation) and in children (median 7.7 years of age)	Early gestation and childhood	Human	Not investigated	Higher levels of PFOS, PFOA, PFDeA associated with higher total cholesterol and/or LDL cholesterol among girls; higher HDL cholesterol with PFAS among boys and girls	Yes	[175]
Levels of PFAS (PFOS, PFOA, PFNA, PFDA, and PFHxS) in 9-year-old children from the European Youth Heart Study	Childhood	Human	Not investigated	Inverse correlation between PFOA, PFDA, PFHxS, and leptin; positive and negative associations between adiponectin and PFOA or PFHxS, respectively, in boys	Yes	[176]
Maternal serum PFOA levels at gestational week 30	Late gestation	Human	Not investigated	Increased risk of overweight or obesity, larger waist circumference, serum insulin, and leptin with PFOA exposure in girls at 20 years of age	Yes	[177]
Maternal PFOA and PFOS levels in serum at gestational age 24 ± 10 weeks	Mid-gestation	Human	Not investigated	Increased risk of waist to height ratio > 0.5 with PFOA or PFOS at 5–9 years of age, with the PFOS association slightly greater in Greenlandic girls than boys	Yes	[178]
Levels of PFOS and PFOA in newborn dried blood spots	Late gestation	Human	Not investigated	Lower BMI with PFOA and PFOS, with stronger associations observed for girls	Yes	[179]
Levels of PFOS and PFOA in maternal plasma during 1st and 2nd trimester, and cord blood	Gestation	Human	Not investigated	Inverse association between maternal PFOA, PFOS, and children’s weight and body mass index in first year of life in boys, with no association observed in girls	Yes	[180]
Levels of PFOA and four other long-chain PFAS in maternal serum during 3rd trimester	Late gestation	Human	Not investigated	Inverse association between PFNA, PFDeA, PFUnDA, PFDoDA, and birth weight in girls; increased odds of small for gestational age with PFDeA, PFUnDA in girls; lower average height z-score with PFDeA, PFUnDA, PFDoDA; reduced childhood height in boys with PFNA, PFDoDA exposure	Yes	[181]
Maternal plasma levels of PFOS and PFOA during 1st and 2nd trimester	Early gestation	Human	Not investigated	No significant associations between maternal PFOS, PFOA and body mass index, risk of overweight, and waist circumference at 7 years of age	Not reported	[182]
Levels of PFOS, PFOA, and PFHxS in maternal serum, median gestational age 15 weeks	Early-mid gestation	Human	Not investigated	Inverse relationship between PFAS and birth weight; positive relationship between PFOA and body weight at 20 months of age	Only females were evaluated	[183]

**Table 6:** developmental PFAS exposure and the epigenome in the context of cardiovascular health

Exposure details	Window of exposure	Model	Epigenetic/molecular effect	Phenotype	Sex specificity	Reference
PFOS and PFOA concentrations in maternal serum	Gestation	Human	In cord blood, 4 differentially methylated CpGs met FDR cutoff; several CpGs showed changes that overlapped between 2 cohorts; 1 differentially methylated region identified met FWER cutoff; several CpGs, regions map to CVD-relevant genes, including *ZFP57, NTN-1*	Not investigated	Not reported	[187]
Cord blood levels of PFOS, PFOA, PFHxS, PFDA, and PFNA	Gestation	Human	In cord blood, 10 598 differentially methylated CpGs with PFOS in males, linked to pathways including cardiac proliferation	Not investigated	Yes—15% of male-specific CpGs linked to X chromosome	[192]
PFOS and PFOA levels in umbilical cord serum	Gestation	Human	Inverse correlation between serum PFOA and global DNA methylation in umbilical cord serum	Not investigated	Not reported	[193]
Concentrations of PFOA, PFOS, PFNA, and PFUA in cord blood	Gestation	Human	Inverse association between PFOS and Alu methylation in cord blood	Not investigated	Not reported	[194]
Levels of PFOA and PFOS in newborn dried blood spots	Gestation	Human	In dried blood spots, >90th percentile PFOA concentration correlated with DNA methylation CpG near SCRT2, SRXN1; >90th percentile PFOS concentration was related to a CpG in *GVIN1* in boys and a CpG in *ZNF26* in girls). Log-scaled, continuous PFOS concentration associated with methylation of CpG in *PTBP1*, a gene associated with cardiovascular disease and development	Not investigated	Yes	[195]

#### Cardiovascular effects of developmental PFAS exposure in animals

Numerous animal studies, conducted in rodents, zebrafish, Xenopus, and chicken embryos, have demonstrated that developmental PFAS exposures are associated with adverse cardiovascular outcomes. PFOA exposure in chicken embryo cardiomyocytes (exposures occurring while in the embryo or ex-vivo) led to reduced viability, altered cell morphology, and de-regulated intracellular calcium levels [148]. Additional work demonstrated that developmental exposure to PFOA, or the PFOA replacement, hexafluoropropylene oxide dimer acid (HFPO-DA, GenX) in chicken embryos led to altered cardiac function and morphology [149, 150]. Developmental PFAS exposures have been shown to cause cardiac developmental defects in Xenopus embryos [151] or in weanling rats [152] or mice [153], although the sex-specific effects of these chemicals were not investigated. At a molecular level, single cell analysis of zebrafish embryos revealed that developmental PFOA exposure disrupts the expression of genes associated with cardiac contractility and differentiation in the cardiac cell population [154]. Additional work has demonstrated that gestational exposure to PFAS alter maternal and offspring lipids, with potential implications for long-term cardiovascular health. Gestational GenX exposure led to altered maternal and offspring glucose and lipid metabolism in rats, although sex-specific effects were unclear [155]. Low-dose developmental PFOA exposure in female mice led to an increased rate of weight gain and an increase in serum leptin and insulin in mid-life [156]. Collectively, these data suggest that PFAS exposures in early life may have adverse cardiovascular consequences. However, given substantial differences in PFAS dosing and toxicokinetics between humans and animals, the drawing of direct parallels between species must be done with caution.

#### Cardiovascular effects of developmental PFAS exposure in humans

Early life PFAS exposures are linked to adverse cardiovascular effects in humans, although some studies have produced conflicting and difficult to interpret findings. Several endpoints have been investigated, including maternal metabolic and cardiovascular effects, as well as offspring congenital heart defects, blood pressure, blood glucose, lipid profiles, levels of hormones regulating appetite and obesity, BMI, and growth. In pregnant women, PFAS exposures are linked to adverse metabolic and cardiovascular effects. Maternal levels of PFAS are positively associated with total cholesterol and triglycerides [157], as well as higher blood glucose and increased risk of gestational diabetes mellitus [158]. PFAS exposures during pregnancy are also associated with an increased risk of hypertension or preeclampsia [159, 160], with differences observed based on the sex of the fetus [160]. Although the implications for the long-term health of the offspring are unclear, adverse effects of maternal diabetes, hyperglycemia, preeclampsia, and high cholesterol on offspring cardiovascular health have been reported in human observational studies and experimental models [14, 15, 161-164].

An increasing number of studies have begun to assess the effects of PFAS on children and adolescents. With regard to PFAS and congenital heart defects in humans, data are limited, with conflicting results. One study found an association between maternal levels of several PFAS and congenital heart defects in both sexes [165], while a second study also found a weak association between PFOA and congenital heart defects [166]. However, other studies have found no association [167, 168]. Effects of PFAS on blood pressure have also been reported, with several studies showing modest increases in blood pressure with PFAS exposure. For example, PFAS have been associated with increased blood pressure in several different populations of children and adolescents, with some studies identifying sex differences [48] and others not [169, 170]. Other studies, however, have observed no effects on blood pressure [171, 172].

PFAS exposures at multiple time points during early life have also been linked to altered blood glucose, lipids, and metabolic homeostasis in children and adolescents. Maternal blood levels of several PFAS were linked to increased lipids in cord blood [173]. Gestational exposure to PFOA and perfluorohexanesulfonic acid (PFHxS) were associated with unfavorable cardiometabolic risk scores in adolescence, driven by altered insulin levels and insulin resistance, blood pressure, waist circumference, and other factors [27]. Several studies have demonstrated that blood PFAS levels in children and adolescents are associated with increased blood glucose and/or alterations in lipids and amino acids [170, 174, 175], with some investigations identifying sex differences [175]. Other researchers found no effects of PFAS on blood lipids or glucose [171]. PFHxS levels in children were associated with altered levels of adipokines, including adiponectin and leptin, with effects on adiponectin found only in boys [176]. Effects of developmental exposure to various PFAS on childhood BMI and growth have been investigated, with PFAS being associated with increased BMI or growth [177, 178], decreased BMI or growth [179-181], no change [182], or effects that differed based on age [183]. Sex differences in the effects of these chemicals on BMI have been reported [177, 180].

Conflicting research findings into the effects of PFAS on cardiovascular health are likely due to differences in the toxicokinetics and toxicodynamics of the various PFAS, the timing of outcome measurement (pre vs. post-puberty), the timing of exposure, the experimental methods employed, sample sizes, as well as lifestyle habits and other confounding factors among the populations studied. Therefore, further investigations into the effects of developmental PFAS on human cardiovascular health are necessary.

#### Effects of developmental PFAS exposure on the epigenome

A relatively modest number of studies have examined the effects of PFAS exposures on the epigenome. Moreover, the effects of PFAS on the cardiac epigenome, and the implications these changes have for cardiovascular disease, have not been investigated. Studies into the epigenetic effects of PFAS have been conducted in cells *in vitro*, in animal models, and in human populations, with the overwhelming majority of the research focused on DNA methylation. Likewise, most studies thus far have focused on legacy PFAS such as PFOA and PFOS, and very little published work to date has examined newer generation PFAS.

Several human studies have demonstrated associations between blood levels of PFAS and DNA methylation at various loci, including LINE-1 elements, which are linked to cardiovascular diseases [184]. Notably, recent work has also linked PFAS exposures to altered expression of miRNAs, where exposure in women was associated with changes in miRNAs that have functions in cardiovascular and Alzheimer’s diseases [185]. Among the predicted target genes for the miRNAs was *DNMT3A*, suggesting potential crosstalk between miRNA and DNA methylation [185]. A second study in this group identified PFAS-associated changes in DNA methylation in several biological pathways, including cardiac hypertrophy [186]. Investigations into the effects of developmental exposure to PFAS on the human epigenome have utilized maternal or offspring blood, cord blood, and blood spots. PFAS levels in maternal serum were associated with changes in DNA methylation in cord blood at several loci implicated in cardiovascular disease and development [187-191]. Additional work has produced similar findings in cord blood global or site-specific DNA methylation [192-194], with some studies identifying sex differences [192] and others finding no evidence of sex specificity [193, 194]. Concentrations of PFOS and PFOA in infant blood spots were associated with sex-specific changes in DNA methylation at a small number of loci, including the gene *PTBP1*, related to cardiovascular disease and development [195].

Several additional studies *in vitro* and in animal models have demonstrated PFAS-induced epigenetic changes; however, few investigations into the effects of PFAS on the cardiac epigenome have been conducted thus far. Some *in vitro* evidence suggests that PFAS exposures modulate the epigenome in undifferentiated cells. PFOS exposure leads to increased oxidative stress, altered expression of DNA methyltransferases and sirtuins, and decreased DNA methylation in human trophoblast cells [196]. Likewise, PFOS exposure altered DNA methylation in differentiating adipocytes [197]. In mouse embryoid bodies, PFOS increased expression of the Polycomb complex members and markers of pluripotency and decreased expression of factors associated with differentiation [198]. Exposure to a mixture of PFAS along with the polychlorinated biphenyl PCB126 led to increased expression of dnmt1 in zebrafish embryos [199]. The effects of these chemicals on cardiac differentiation *in vitro* or *in vivo*, however, have not been investigated.

### Developmental phthalate exposure and cardiovascular health

Phthalates are a large class of chemicals that are divided into two groups based on their molecular weight and chemical properties. High molecular weight phthalates are found in medical tubing, food packaging, vinyl toys, and building products, while low molecular weight phthalates are found in personal care products such as perfumes, lotions, nail polish, and shampoos [200]. Human exposure to phthalates occurs through inhalation, skin absorption, ingestion, and intravenous injection [200]. Phthalate exposures are linked to a wide array of adverse metabolic, neurodevelopmental, reproductive, and cardiovascular health effects [201]. A summary of the cardiovascular and epigenetic effects of developmental phthalate exposures can be found in Tables 7 and 8, respectively.

**Table 7:** developmental phthalate exposure and cardiovascular health

Exposure details	Window of exposure	Model	Epigenetic/molecular effect	Phenotype	Sex specificity	Reference
Exposure to DEHP (500 mg/kg) during pregnancy	Embryonic days 6.5–14.5	Mouse	Not investigated	Reduced fetal weight, increased septal defects, and ventricular myocardium noncompaction	Not reported	[202]
DEHP (0.02 pg) injected into the yolk sac of the embryo	1–4 days post-fertilization	Zebrafish	Not investigated	Increased mortality at 2 and 3 days post-fertilization; reduced heart rate at 3 and 4 days post-fertilization; pericardial edema and heart looping disorder 3 days post-fertilization	Not reported	[203]
Benzyl butyl phthalate (0, 0.3, 1.9, and 3.8 μM) in petri dish water	4–72 h post-fertilization	Zebrafish	Not investigated	Dose-dependent cardiac malformations (pericardial edema, elongated and string-shaped heart, looping abnormalities), reduced heart rate	Not reported	[204]
Dibutyl phthalate (0, 0.36, 1.8, and 3.6 μM) in petri dish water	4–72 h post-fertilization	Zebrafish	Not investigated	Dose-dependent increase in pericardial edema, structural abnormalities; reduced heart rate at highest dose	Not reported	[205]
DEHP administered to dams (0, 1, 10, and 100 mg/kg/day via oral gavage)	Lactation—postpartum days 1–21	Rat	Not investigated	Dose-dependent increased fasting blood glucose; decreased insulin receptor expression and insulin receptor substrate phosphorylation in female hearts at postnatal day 60	Only females were evaluated	[206]
DEHP administered to dams (1, 10, and 100 mg/kg/day) or olive oil control by oral gavage	Lactation—postpartum days 1–21	Rat	Not investigated	Increased blood glucose, decreased glucose uptake and oxidation, and decreased insulin receptor expression in male hearts at post-natal day 22	Only males were evaluated	[207]
DEHP (1, 10, 100, and 300 mg/kg) administered intragastric under anesthesia	5–6 weeks of age, for a period of 35 days	Mouse	Not investigated	Inhibition of fatty acid beta oxidation and TCA cycle; increased glycolysis; inhibition of synthesis and transport of fatty acids in hearts of male mice	Only males were evaluated	[208]
DEHP (50 and 100 μg/mL) or DMSO vehicle in culture medium	72 h	Neonatal rat cardiomyocytes from 1-day-old rats	Not investigated	Increase in cardiomyocyte fatty acid transport and beta oxidation; increased expression of genes associated with fatty acid transport and beta oxidation	Not reported	[209]
DEHP (300 mg/kg/day) by oral gavage	Gestational day 14 until birth	Rat	Not investigated	Decreased activity in post-natal day 60 offspring; decreased activity and blood pressure in post-natal day 200 offspring	Only males were evaluated	[210]
Phthalate exposure estimated via job exposure matrix	Reported occupational exposure during periconceptional period	Human	Not investigated	Maternal phthalate exposure associated with perimembranous ventricular septal defect, patent ductus arteriosus, secundum atrial septal defect, and pulmonary valve stenosis; paternal exposures associated with perimembranous ventricular septal defect and pulmonary valve stenosis	Not reported	[215]
Phthalate exposure estimated via job exposure matrix	Reported occupational exposure during periconceptional period	Human	Not investigated	Paternal phthalate exposure associated with increased incidence of congenital heart defects; no significant associations observed for maternal exposures	Not reported	[216]
Maternal urinary phthalate metabolites in 1st, 2^nd^, and 3rd trimester	Gestation	Human	Not investigated	Increased phthalic acid in 1st trimester associated with increased pericardial fat index at 10 years of age	Effects observed in both sexes	[217]
Maternal urinary phthalates in 1st, 2^nd^, and 3rd trimester		Human	Not investigated	3rd trimester high molecular weight phthalates associated with lower systolic and diastolic blood pressure in girls (mean age 9.7 years); no associations observed for boys	Yes	[46]
Urinary phthalate metabolites in children 6–8 years of age	Childhood	Human	Not investigated	Significant positive association between several phthalate metabolites and blood pressure *z*-score, pulse pressure, mean arterial pressure; monomethyl phthalate associated with increased risk of high blood pressure	Not reported	[218]
Urinary metabolite concentrations of three phthalates (low molecular weight, high molecular weight, and DEHP)	Childhood, adolescence—ages 6–19	Human	Not investigated	Positive association between metabolites of DEHP and systolic blood pressure	Not reported	[219]
Maternal urinary phthalate concentrations in 1st, 2nd, and 3rd trimester	Gestation	Human	Blood leukocyte DNA methylation at *H19* or *HSD11B2* do not mediate association between phthalate exposure and adiposity	1st and 2nd trimester phthalate metabolites positively (MBP and MIBP) or negatively (MBzP) associated with adiposity in adolescent girls; 2nd trimester MBzP associated with increased BMI and waist circumference in boys	Yes	[220]
Maternal urinary phthalate metabolites at 13 and 26 weeks gestation	Early-mid gestation	Human	Not investigated	Early gestational phthalate metabolites (monobenzyl, monocarboxyoctyl, and monocarboxynonyl phthalate) negatively associated with 8-isoprostane at 9 years of age; early gestational mono(2-ethylhexyl) and mono(2-ethyl-5-carboxypentyl) phthalate positively associated with 8-isoprostane at 14 years of age	Not reported	[221]

**Table 8:** Developmental phthalate exposure and the epigenome in the context of cardiovascular health

Exposure details	Window of exposure	Model	Epigenetic/molecular effect	Phenotype	Sex specificity	Reference
Maternal urinary phthalate concentrations at 13 and 26 weeks gestation	Early-mid gestation	Human	Monoethyl phthalate levels in early and mid-gestation associated with Alu repeat methylation in cord blood; association with LINE-1 methylation present but weaker; increased concentration of di-(2-ethylhexyl) phthalate metabolites mid gestation associated with reduced methylation of Alu repeats in blood at 9 years of age	Not investigated	Not reported	[223]
Maternal urinary phthalate concentrations during 3rd trimester	Late gestation	Human	Negative association between placental LINE-1 methylation and concentrations of urinary phthalate metabolites (MEHHP and sum of DEHP)	Significant positive association between concentrations of several metabolites MEHHP, MEOHP, sum of DEHP, and fetal growth restriction	Not reported	[224]
1st trimester urinary concentrations of 11 phthalate metabolites	Early gestation	Human	Significant inverse association between placental *H19* methylation and the sum of phthalate metabolites and low molecular weight phthalate metabolites; concentrations of sum and low molecular weight metabolites inversely associated with methylation of *IGF2DMR0* in placenta	No significant effects of DNA methylation or imprinted gene expression on birth length or weight	Yes—Altered methylation of *IGF2DMR0* with phthalate exposure more likely to occur in female placenta	[227]
Maternal urinary phthalate concentrations from patients with or without gestational diabetes mellitus in 2nd trimester	Mid gestation	Human	In maternal serum, increased miR-16-5p expression with MBzP levels; increased miR-29a-3p expression with adjusted MEHP; negative association between miR-29a-3p expression and unadjusted and adjusted MBP concentration; negative association between miR-29a-3p expression and unadjusted MiBP concentration	MEHP levels higher in non-diabetic patients compared to gestational diabetes	Not reported	[230]
DEHP (5, 50, 100 µg/mL) or DMSO vehicle	5 days, followed by differentiation into cardiomyocytes	Mouse P19 embryonic carcinoma cell line	In P19 cells, increased expression of *Dnmt1, Dnmt3a* at higher doses; increased methylation of CpGs within *Ppara* and *Pparg1* genes	Increased rate of differentiation; increased beat rate	Not applicable	[231]
DEHP in chow (25 mg DEHP/kg chow in 7% corn oil)	2 weeks prior to mating, through gestation and lactation	Mouse	In mouse heart, sex-specific changes in DNA methylation at hundreds of CpGs and regions at 5 months of age; several genes with differential DEHP-induced methylation were also differentially methylated in heart samples from patients with heart failure	Not investigated	Yes	[232]
DEHP (0, 1, 10, and 100 mg/kg per day) or olive oil vehicle via oral gavage	Gestation days 9-21	Rat	Increased *Hdac2*, *Dnmt1*, *Dnmt3a*, and *Dnmt3b* protein expression, increased global DNA methylation in gastrocnemius in both sexes	Elevated blood glucose, insulin resistance, reduced insulin receptor, and reduced glucose uptake and oxidation in gastrocnemius muscle at 2 months of age in both sexes	Effects observed in both sexes	[234]
DEHP or DBP (50, 250 μg/l) or acetone vehicle	Starting 1.5–1.7 h post-fertilization and lasting 4 days	Zebrafish	In embryos, hypomethylation of *nppa* and *ctnt* genes related to cardiac development and upregulation of gene expression; hypermethylation of *tbx5b* gene and down-regulation of expression with highest DBP dose	Increased heart rate, pericardial edema, apoptosis	Not reported	[235]

#### Cardiovascular effects of developmental phthalate exposure in animals

Animal and *in vitro* studies provide substantial evidence that phthalate exposures are deleterious to cardiovascular health. In mice, maternal exposure to diethylhexyl phthalate (DEHP) caused cardiac developmental defects [25, 202]. Work in zebrafish yielded similar findings, where exposure to DEHP [203], butyl benzyl phthalate (BBP) [204], or dibutyl phthalate (DBP) [205] during development led to cardiac developmental defects. However, whether there are sex differences in phthalate-induced developmental defects is unclear. Additional work has linked lactational exposure to DEHP to systemic and cardiac-specific changes in insulin signaling in female offspring rats [206] and in male offspring [207], and young male mice exposed to DEHP developed cardiac mitochondrial dysfunction [208]. These *in vivo* changes in metabolism have also been observed *in vitro*, where DEHP exposure in neonatal rat cardiomyocytes led to shifts in cellular metabolism associated with cardiac disease states [209]. Developmental exposure to DEHP led to decreased blood pressure in adult male rats, suggesting that DEHP can cause long-term changes in cardiovascular physiology [210]. Finally, work in cell lines and animal tissue demonstrates that phthalates interfere with synthesis of prostaglandins, lipid signaling molecules with important roles in cardiovascular health [211].

#### Cardiovascular effects of developmental phthalate exposure in humans

In human epidemiologic studies, phthalate exposures in adults are linked to a variety of adverse cardiovascular outcomes, including decreased heart rate variability, coronary heart disease, and atherosclerosis [212-214]. Likewise, a number of human studies have demonstrated associations between early life phthalate exposure and cardiovascular diseases. First, several investigations have linked phthalates to congenital heart defects. Phthalate exposure during pregnancy cooperated with genetic factors to increase the risk of congenital heart defects in a population of Chinese children [26]. Maternal or paternal [215] or only paternal [216] occupational exposures to phthalates were associated with an increased risk of congenital heart defects. Sex-specific effects of phthalate exposures on congenital heart defects are unclear, as they were not investigated in these studies. Notably, assessments of exposure were based on questionnaires rather than measurement of phthalate levels in biological samples, so further investigation is warranted to confirm these findings. An additional investigation demonstrated that maternal urinary phthalate levels were associated with a significant increase in pericardial fat in children at 10 years of age, with no sex differences observed [217], suggesting that phthalates may alter fat deposition in organs.

Phthalate exposures have also been correlated with changes in blood pressure. Maternal urinary phthalate levels were associated with decreased blood pressure in female children [46]. However, additional studies that measured phthalate concentrations in the urine of children found that metabolites of several phthalates were associated with increased blood pressure in children and adolescents [218, 219]. Discrepancies in the findings are likely due to differences in the timing of exposure or outcome measurement (maternal vs. offspring), distinct toxicokinetics and toxicodynamics of the various phthalates, different doses and windows of exposure, as well as variations in genetics, diet, lifestyle and other factors of the populations investigated.

Additional work demonstrated associations between phthalate exposures and adiposity in children that were dependent upon sex, the specific phthalate, and the timing of exposure. In girls, their mothers’ urinary levels of phthalate metabolites during pregnancy were associated with increased adiposity in peri-adolescence [220]. Whether these changes in body composition persist and the long-term implications of these findings for cardiovascular health are unclear. Lastly, prenatal phthalate exposures have been linked to age-dependent alterations in levels of 8-isoprostane, a marker of oxidative stress associated with CVD, in the blood of children and adolescents [221].

Findings in humans and animals thus collectively provide compelling evidence that phthalate exposures during early life are linked to adverse cardiovascular effects. However, more work is necessary to determine the sex-specific effects of these chemicals, as well as the effects of developmental phthalate exposures on cardiovascular health across the life course, including middle and old age where cardiovascular diseases are most prevalent. Moreover, as most research thus far has focused only on DEHP, further investigation into other phthalates is warranted.

#### Effects of developmental phthalate exposure on the epigenome

Numerous human, animal, and *in vitro* studies demonstrate that phthalate exposures are associated with changes in the epigenome. Similar to Pb and PFAS, the majority of the studies thus far have focused on DNA methylation, and few studies have investigated the effects of phthalates on the epigenome specifically in the cardiovascular system. Phthalate-induced epigenetic changes have been reported for both early life and adult exposures. In adolescent and young adult Taiwanese, urinary metabolites of DEHP were associated with an increase in carotid intima-media thickness (CIMT), a marker of subclinical atherosclerosis, as well as increased global DNA methylation, and the authors proposed that DNA methylation may mediate the effect of DEHP on CIMT. Although both males and females were included in the study, sex differences were not investigated [222].

Several studies have linked maternal phthalate exposures to altered DNA methylation at repetitive elements. Maternal urine levels of monoethyl phthalate or DEHP were associated with decreased DNA methylation at Alu repetitive elements in cord blood or in the blood of children at 9 years of age, respectively [223], or with decreased LINE-1 methylation in the placenta and fetal growth restriction [224]. Sex differences were not reported in either study. Although the implications of these findings for cardiovascular health are unclear, methylation of Alu and LINE-1, as well as fetal growth restriction, has all been associated with CVD [225, 226]. Prenatal phthalate exposure has also been associated with sex-specific dysregulation of DNA methylation and expression of the imprinted genes *H19* and *IGF2* [227], both of which have important roles in cardiovascular disease and development [228, 229]. Additional work has identified a link between maternal exposure to several phthalates and alterations in serum miRNA levels linked to gestational diabetes [230], a condition linked to increased risk of cardiovascular disease [162].

Additional work *in vitro* and in animals corroborates the findings in humans. *In vitro*, exposure of P19 cells to DEHP prior to cardiac differentiation led to altered expression of *DNMT1* and *DNMT3A* before and during differentiation [231]. As in humans, few studies have investigated the effects of early life phthalate exposures specifically on the cardiovascular epigenome. Recent work from our lab demonstrated that maternal exposure to DEHP during gestation and lactation led to sex-specific changes in DNA methylation in the hearts of offspring mice at 5 months of age, long after cessation of exposure [232]. Many of the genes differentially methylated after DEHP exposure also exhibited differential methylation in cardiac tissue from human heart failure patients, suggesting that DEHP exposure alters DNA methylation at disease-relevant genes [232]. Maternal exposure to a mixture of plastics including phthalates led to sex and dose-specific alterations in puberty onset, including early onset puberty in F3 females, as well as obesity in F3 males and females [82]. Although cardiovascular outcomes were not specifically investigated in this study, precocious puberty is a phenomenon associated with obesity and cardiovascular disease in humans [233]. In rats, exposure to DEHP during pregnancy led to increased global DNA methylation and expression of Dnmt1, Dnmt3a, and Dnmt3b transcript and protein in the gastrocnemius muscle of both male and female offspring, concomitant with altered systemic glucose homeostasis [234]. The cardiovascular implications of this work are unclear; however, systemic glucose homeostasis is closely coupled to cardiovascular health [9]. Epigenetic effects of phthalates have also been demonstrated in zebrafish, where DEHP and di-*n*-butyl phthalate (DBP) exposure led to altered DNA methylation of transcription factors critical for normal cardiac development, concomitant with cardiac developmental defects [235].

### Multi- and transgenerational effects of Pb, PFAS and phthalates on cardiovascular health

To date, there have only been a small number of studies that have investigated multi- or transgenerational effects of exposure to Pb, PFAS, or phthalates. A summary of the studies outlined below can be found in Table 9.

**Table 9:** Multi- and transgenerational effects of Pb, PFAS, and phthalates

Factor	Window of exposure	Model	Epigenetic/molecular effect	Phenotype	Sex specificity	Reference
Pb, measured in maternal (reflecting grandmother’s exposure) and child (reflecting mother’s exposure) neonatal dried blood spots, as well as child blood	Gestation	Human	Altered DNA methylation in the blood of grandchildren at genes associated with cardiovascular development and disease (*NDRG4, APOA5, NINJ2*, and *TRPV2*)	Not investigated	Not reported	[128]
F0 generation exposed as embryos to Pb(NO_3_)_2_) in water at 10 μM	<2 h post-fertilization until 24 h	Zebrafish	In embryos, differential expression of genes involved in epigenetic modifications in F2 generation; genes associated with CVD significantly differentially expressed in F2 females	Not investigated	Yes	[240]
100 ppm Pb acetate dissolved in distilled deionized drinking water	2 months prior to mating	Mouse	Altered DNA methylation of *Th* in the cortex in F3 females; altered DNA methylation of *Bdnf* in the hippocampus of F3 males	Reduced body weight; decreased corticosterone in F3 females	Yes	[241]
PFBS (0, 1.0, 2.9, and 9.5 μg/l) exposure in F0 generation	Embryo stage until reaching sexual maturity (6 months of age)	Zebrafish	Reduced global DNA methylation levels in F1 embryos	Decreased weight in F1 generation eggs; increased weight of F2 eggs	Yes—effects on F0 female reproduction	[243]
PFBS (0.0005, 0.01, 0.1, 0.5, 1, and 2 mM—equivalent to 0.15, 3.0, 30.0, 150, 300, and 600 mg/l) in growth medium	L4 stage larvae were exposed for 48 h	*C. elegans*	Not investigated	Movement defects in F1 generation; reduced lifespan in F4 and F5 generations continuously exposed to low-dose PFBS	Not reported	[244]
PFBS and PFHxS in growth medium (1 ng/l)	Egg stage, for 72 h	*C. elegans*	In larvae, altered expression of enzymes and genes important for lipid metabolism	Not investigated	Not reported	[70]
6:2 chlorinated polyfluorinated ether sulfonate (F-53B, 0, 5, 50, or 500 μg/l)	180 days	Zebrafish	Altered expression of thyroid axis genes in F1 and F2 embryos/larvae	Increased T4 levels in F1 embryos; increased mortality, impaired swim bladder formation, increased T3 and T4 levels in F1 larvae; impaired swim bladder formation in F2 larvae	Not reported	[245]
Levels of PFAS in serum from partners of pregnant women	Paternal exposure	Human	Negative association between PFNA and global sperm DNA methylation in all populations combined; positive association between PFOA and LINE-1 methylation in Kharkiv; negative association between PFHxS and sperm global DNA methylation in Greenland; negative association between PFOS and sperm global DNA methylation in Warsaw; positive association between PFOS and satellite alpha repeat methylation in Kharkiv	Not investigated	Only males were evaluated	[247]
Di-n-butyl phthalate (0 and 500 mg/kg) or corn oil vehicle via oral gavage	Gestational days 8–14	Rat	Increased levels of betaine and betaine homocysteine S-methyltransferase, global DNA hypomethylation in F1 and F3 testis at 60 days of age	Decreased sperm counts in F1–F3 generation at 60 days of age	Only males were evaluated	[248]
DEHP (500 mg/kg) or corn oil vehicle by oral gavage	Gestational days 7–14	Mouse	Not investigated	Decreased sperm count and mobility in F1–F4 generations; impaired spermatogonial stem cell function in F3	Only males were evaluated	[249]
DEHP (20 and 200 µg/kg/day and 500 and 750 mg/kg/day) or corn oil control by oral gavage	Gestational day 10.5 until birth	Mouse	Not investigated	Accelerated puberty, disruptions in estrous cycle, and decreased markers of fertility in F1 and F2; increased litter size in F2; accelerated puberty, disruptions in estrous cycle and decreased anogenital distance in female offspring	Only females were evaluated	[250]
DEHP (1, 20, 50, or 300 mg of DEHP/kg/day) or oil control by oral gavage	Gestational day 14 until birth	Rat	Not investigated	Decreased rate of pregnancy, lower body weight, increased litter size, and decreased pup weight in F3	Only females were evaluated	[251]
Phthalate mixture: 35% DEP, 21% DEHP, 15% DBP, 8% DiBP, 15%DiNP, and 5% BzBP (20 μg/kg–500 mg/kg) or corn oil vehicle via oral gavage	Gestational day 10 until birth	Mouse	Not investigated	Decreased testosterone and inhibin B, increased follicle stimulating hormone and luteinizing hormone in F1; lower testosterone and reduced percentage of antral follicles in F2; altered estrous cycle, increased ovarian weight, decreased luteinizing hormone, altered numbers of follicles in F3	Only females were evaluated	[252]
DEHP (150 and 200 mg/kg) or corn oil control daily via oral gavage	Gestational days 7–14	Mouse	Higher pituitary *Gnas* expression with stress and ancestral DEHP exposure in both F3 sexes	Lower corticosterone concentrations after stress in F3 females; dose-dependent behavioral changes and reduced seminal vesicle weights in F3 males	Yes	[254]
DEHP (0 and 40 μg DEHP/kg) or 0.1% DMSO vehicle daily via oral gavage	From 0.5 to 18.5 days after mating	Mouse	Reduced DNA methylation in *Igf2r* and *Peg3* regulatory regions in F1 male and female primordial germ cells and in F2 oocytes	Not investigated	Yes	[256]

#### Pb exposure

There are currently no studies that have investigated multi- or transgenerational effects of Pb exposure on cardiovascular tissues. However, a small number of studies have identified epigenetic effects on genes linked to cardiovascular development and disease in other tissues. Using neonatal bloodspots in humans, grandmothers’ exposure to Pb was associated with altered DNA methylation in the blood of grandchildren, with alterations occurring at several genes (*NDRG4, APOA5, NINJ2*, and *TRPV2*) with important functions in cardiovascular development and disease [128, 236-239]. In zebrafish, ancestral exposure to Pb resulted in neurobehavioral deficits as well as sex-specific, differential expression of genes involved in epigenetic regulation [240]. Notably, in females, cardiovascular disease-related genes were also significantly enriched among differentially expressed genes [240]. In mice, prenatal Pb exposure led to smaller F3 offspring size in addition to altered corticosterone levels in F3 females [241]. Both males and females showed region-specific alterations in DNA methylation in the brain [241]. Cardiovascular health outcomes and DNA methylation were not investigated; however, the findings of altered size at birth and glucocorticoid levels, both associated with cardiovascular disease in humans [226, 242], suggest that cardiovascular effects should be investigated in future studies.

#### PFAS exposures

Few investigations into the multi- or transgenerational effects of PFAS exposures have been conducted to date. Among the small number of studies available, the majority have been performed in insect and worm species (summarized in Table 9). Exposure to perfluorobutane sulfonate (PFBS), a replacement for PFOS, led to transgenerational effects on reproductive function in fish [243], as well as movement defects in the F1 generation of *Caenorhabditis elegans* [244]. Continuous exposure to low-dose PFBS in worms ancestrally exposed to PFBS resulted in significantly shortened lifespan in F4 and F5 generations [244]. PFHxS and PFBS also caused transgenerational alterations in lipid metabolism pathways in *C. elegans* [70]. In zebrafish, ancestral F-53B exposure led to altered expression of several thyroid hormone regulated genes, a trend toward increased thyroxine levels, as well as impaired swim bladder inflation in animals not directly exposed to the chemical [245]. Epigenetic effects were not investigated in any of these studies.

The effects of PFAS on the germline epigenome are also poorly understood. In mice, exposure to PFOA led to significant alterations in several miRNAs in the testes [246]. In a more recent human study, an investigation into the effects of PFAS on sperm DNA methylation in men from three regions in Europe and the Arctic yielded inconclusive results [247]. No consistent changes in global DNA methylation were observed with PFAS exposures; however, small but statistically significant changes in DNA methylation were observed with specific PFAS in specific populations [247]. Moreover, the study only included partners of pregnant women, potentially selecting for men with healthier reproductive function [247]. Given the paucity of investigations into the germline and transgenerational effects of PFAS in mammals, more research in this area is urgently needed.

#### Phthalate exposures

Compared to Pb and PFAS, more studies have been conducted into the multi and transgenerational effects of phthalates, although they have focused primarily on hormonal functions, with effects observed in both males and females. In males, studies in rats and mice demonstrated that exposure to DEHP or DBP during pregnancy led to reduced sperm counts and mobility [248, 249], as well as impaired spermatogonial stem cell function [249]. Altered sperm function in F3 rats was accompanied by lower global levels of DNA methylation [248]. As noted previously in rats, maternal exposure to a mixture of plastics (BPA and phthalates) led to reproductive alterations and obesity in F3 animals of both sexes [82].

In females, separate studies with rats and mice showed that ancestral exposure to DEHP had adverse effects on reproductive function in F3 animals, although whether epigenetic mechanisms played a role in these effects were not investigated [250, 251]. Exposure of pregnant mice to a mixture of six phthalates led to premature reproductive aging in F3 females [252], a phenomenon associated with cardiovascular diseases in humans [253]. Ancestral exposure to DEHP led to female-specific changes in levels of corticosterone in response to stress, as well as altered expression of the imprinted gene, *Gnas* [254], which has important functions in cardiovascular health [131, 255]. Further work showed multigenerational effects of DEHP exposure on DNA methylation at imprinted loci in oocytes [256], suggesting that these chemicals may have effects on germ cell function across multiple generations.

Although transgenerational effects of phthalates on cardiovascular outcomes have not been investigated, the well-established links between hormonal dysregulation and cardiovascular health [242, 253, 257] suggest that phthalates may also affect cardiovascular health across generations. This important question should be addressed in future studies.

## Conclusion

Although environmental factors play a critical role in the etiology of cardiovascular diseases, how their effects differ by sex is poorly understood. Likewise, future studies are needed to better understand how toxicant exposures impact cardiovascular health across generations (Fig. 2). Indeed, although the studies outlined in this review and documented in Tables 1–9 demonstrate that chemical exposures during early development can have adverse cardiovascular health effects, the precise window(s) of vulnerability are still unclear. Systematic investigation into this question, testing the effects of chemical exposures during different stages of development and during other potentially vulnerable points across the life course (childhood, adolescence, and reproductive senescence) is necessary to gain a deeper understanding of the threat posed by environmental toxicants to cardiovascular health.

This need for additional insight into the adverse generational and sex-specific health effects of chemicals is exemplified by Pb, PFAS, and phthalates. Of note, among the studies identified in Tables 3–9 addressing the cardiovascular, epigenetic, and transgenerational effects of these toxicants, the vast majority do not provide an analysis of sex specificity. Although research practices have become more inclusive, in part through guidance from the National Institutes of Health [258], the importance of sex as a biological variable is still underappreciated in the scientific community at large [259]. Of the tens of thousands of chemicals registered for use in the USA, only a minority have undergone government safety evaluations, highlighting the significant burden on consumers and researchers to elucidate their potential health effects. Given the profound economic and health burden posed by cardiovascular diseases, more research into this important area of public health is urgently needed.

## Data Availability

Not applicable.

## References

[1] Barker DJ . The origins of the developmental origins theory. *J Intern Med* 2007;261:412–7. 17444880 10.1111/j.1365-2796.2007.01809.x

[2] Thornburg KL . The programming of cardiovascular disease. *J Dev Orig Health Dis* 2015;6:366–76. 26173733 10.1017/S2040174415001300 PMC7587080

[3] Peng J , Meng Z, Zhou S et al. The non-genetic paternal factors for congenital heart defects: a systematic review and meta-analysis. *Clin Cardiol* 2019;42:684–91. 31073996 10.1002/clc.23194 PMC6605632

[4] Ornellas F , Carapeto PV, Aguila MB et al. Sex-linked changes and high cardiovascular risk markers in the mature progeny of father, mother, or both father and mother consuming a high-fructose diet. *Nutrition* 2020;71:110612. 10.1016/j.nut.2019.110612 31785517

[5] Reuben A . Childhood lead exposure and adult neurodegenerative disease. *J Alzheimers Dis* 2018;64:17–42. 29865081 10.3233/JAD-180267 PMC6454899

[6] Chamorro-Garcia R , Diaz-Castillo C, Shoucri BM et al. Ancestral perinatal obesogen exposure results in a transgenerational thrifty phenotype in mice. *Nat Commun* 2017;8:2012. 10.1038/s41467-017-01944-z PMC5722856 29222412

[7] Wang Q , Trevino LS, Wong RLY et al. Reprogramming of the epigenome by MLL1 links early-life environmental exposures to prostate cancer risk. *Mol Endocrinol* 2016;30:856–71. 27219490 10.1210/me.2015-1310 PMC4965842

[8] Powell-Wiley TM , Poirier P, Burke LE et al. Obesity and cardiovascular disease: a scientific statement from the American Heart Association. *Circulation* 2021;143:e984–e1010. 33882682 10.1161/CIR.0000000000000973 PMC8493650

[9] Bartnik M , Norhammar A, Ryden L. Hyperglycaemia and cardiovascular disease. *J Intern Med* 2007;262:145–56. 17645583 10.1111/j.1365-2796.2007.01831.x

[10] Ravelli AC et al. Cardiovascular disease in survivors of the Dutch famine. *Nestle Nutr Workshop Ser Pediatr Program* 2005;55:183–91. 10.1159/000082602 16632935

[11] Susser M , Stein Z. Timing in prenatal nutrition: a reprise of the Dutch Famine Study. *Nutr Rev* 1994;52:84–94. 8015751 10.1111/j.1753-4887.1994.tb01395.x

[12] Ravelli AC , van der Meulen JH, Osmond C et al. Obesity at the age of 50 y in men and women exposed to famine prenatally. *Am J Clin Nutr* 1999;70:811–6. 10539740 10.1093/ajcn/70.5.811

[13] Sun C , Burgner DP, Ponsonby A-L et al. Effects of early-life environment and epigenetics on cardiovascular disease risk in children: highlighting the role of twin studies. *Pediatr Res* 2013;73:523–30. 23314296 10.1038/pr.2013.6

[14] Ahmed A , Liang M, Chi L et al. Maternal obesity persistently alters cardiac progenitor gene expression and programs adult-onset heart disease susceptibility. *Mol Metab* 2021;43:101116. 10.1016/j.molmet.2020.101116 PMC7720025 33212270

[15] Hachisuga M , Oki S, Kitajima K et al. Hyperglycemia impairs left-right axis formation and thereby disturbs heart morphogenesis in mouse embryos. *Proc Natl Acad Sci USA* 2015;112:E5300–7. 26351675 10.1073/pnas.1504529112 PMC4586861

[16] Ferey JLA , Boudoures AL, Reid M et al. A maternal high-fat, high-sucrose diet induces transgenerational cardiac mitochondrial dysfunction independently of maternal mitochondrial inheritance. *Am J Physiol Heart Circ Physiol* 2019;316:H1202–10. 30901280 10.1152/ajpheart.00013.2019 PMC6580388

[17] Nascimento L , Freitas CM, Silva-Filho R et al. The effect of maternal low-protein diet on the heart of adult offspring: role of mitochondria and oxidative stress. *Appl Physiol Nutr Metab* 2014;39:880–7. 24905448 10.1139/apnm-2013-0452

[18] Jenkins NDM , Rogers EM, Banks NF et al. Childhood psychosocial stress is linked with impaired vascular endothelial function, lower SIRT1, and oxidative stress in young adulthood. *Am J Physiol Heart Circ Physiol* 2021;321:H532–41. 34328346 10.1152/ajpheart.00123.2021 PMC8461842

[19] Dong M , Giles WH, Felitti VJ et al. Insights into causal pathways for ischemic heart disease: adverse childhood experiences study. *Circulation* 2004;110:1761–6. 15381652 10.1161/01.CIR.0000143074.54995.7F

[20] Carroll JE , Gruenewald TL, Taylor SE et al. Childhood abuse, parental warmth, and adult multisystem biological risk in the coronary artery risk development in young adults study. *Proc Natl Acad Sci U S A* 2013;110:17149–53. 24062432 10.1073/pnas.1315458110 PMC3800991

[21] Tanwar V , Gorr MW, Velten M et al. In utero particulate matter exposure produces heart failure, electrical remodeling, and epigenetic changes at adulthood. *J Am Heart Assoc* 2017;6:e005796. 10.1161/JAHA.117.005796 PMC5533043 28400369

[22] Davuljigari CB , Gottipolu RR. Late-life cardiac injury in rats following early life exposure to lead: reversal effect of nutrient metal mixture. *Cardiovasc Toxicol* 2020;20:249–60. 31541351 10.1007/s12012-019-09549-2

[23] Farzan SF , Howe CG, Chen Y et al. Prenatal lead exposure and elevated blood pressure in children. *Environ Int* 2018;121:1289–96. 30389381 10.1016/j.envint.2018.10.049 PMC6279470

[24] Hudson KM , Belcher SM, Cowley M. Maternal cadmium exposure in the mouse leads to increased heart weight at birth and programs susceptibility to hypertension in adulthood. *Sci Rep* 2019;9:13553. 10.1038/s41598-019-49807-5 PMC6753073 31537853

[25] Tang C , Deng Y, Duan H et al. The effect of maternal exposure to di-(2-ethylhexyl)-phthalate on fetal cardiac development in mice. *J Appl Toxicol* 2018;38:834–42. 29377175 10.1002/jat.3591

[26] Wang C , Xie L, Zhou K et al. Increased risk for congenital heart defects in children carrying the ABCB1 gene C3435T polymorphism and maternal periconceptional toxicants exposure. *PLOS One* 2013;8:e68807. 10.1371/journal.pone.0068807 PMC3714281 23874772

[27] Li N , Liu Y, Papandonatos GD et al. Gestational and childhood exposure to per- and polyfluoroalkyl substances and cardiometabolic risk at age 12 years. *Environ Int* 2021;147:106344. 10.1016/j.envint.2020.106344 PMC7856172 33418195

[28] La Merrill M , Cirillo PM, Terry MB et al. Prenatal exposure to the pesticide DDT and hypertension diagnosed in women before age 50: a longitudinal birth cohort study. *Environ Health Perspect* 2013;121:594–9. 23591545 10.1289/ehp.1205921 PMC3673196

[29] Troisi R , Titus L, Hatch EE et al. A prospective cohort study of prenatal diethylstilbestrol exposure and cardiovascular disease risk. *J Clin Endocrinol Metab* 2018;103:206–12. 29069384 10.1210/jc.2017-01940 PMC5761490

[30] Zhou W , Young JL, Men H et al. Sex differences in the effects of whole-life, low-dose cadmium exposure on postweaning high-fat diet-induced cardiac pathogeneses. *Sci Total Environ* 2022;809:152176. 10.1016/j.scitotenv.2021.152176 PMC11871371 34875320

[31] Fuller CH , Appleton AA, Bulsara PJ et al. Sex differences in the interaction of short-term particulate matter exposure and psychosocial stressors on C-reactive protein in a Puerto Rican cohort. *SSM Popul Health* 2019;9:100500. 10.1016/j.ssmph.2019.100500 PMC6831870 31709298

[32] Lind PM , Salihovic S, van Bavel B et al. Circulating levels of perfluoroalkyl substances (PFASs) and carotid artery atherosclerosis. *Environ Res* 2017;152:157–64. 27771570 10.1016/j.envres.2016.10.002

[33] Pesatori AC , Zocchetti C, Guercilena S et al. Dioxin exposure and non-malignant health effects: a mortality study. *Occup Environ Med* 1998;55:126–31. 9614398 10.1136/oem.55.2.126 PMC1757550

[34] Veenema R , Casin KM, Sinha P et al. Inorganic arsenic exposure induces sex-disparate effects and exacerbates ischemia-reperfusion injury in the female heart. *Am J Physiol Heart Circ Physiol* 2019;316:H1053–64. 30822117 10.1152/ajpheart.00364.2018 PMC6580384

[35] Zuo L , Chen L, Chen X et al. Pyrethroids exposure induces obesity and cardiometabolic diseases in a sex-different manner. *Chemosphere* 2022;291:132935. 10.1016/j.chemosphere.2021.132935 34798107

[36] Liakos M , Parikh PB. Gender disparities in presentation, management, and outcomes of acute myocardial infarction. *Curr Cardiol Rep* 2018;20:64. 10.1007/s11886-018-1006-7 29909444

[37] Regitz-Zagrosek V , Kararigas G. Mechanistic pathways of sex differences in cardiovascular disease. *Physiol Rev* 2017;97:1–37. 27807199 10.1152/physrev.00021.2015

[38] Choi HM , Kim HC, Kang DR. Sex differences in hypertension prevalence and control: analysis of the 2010-2014 Korea National Health and Nutrition Examination Survey. *PLOS One* 2017;12:e0178334. 10.1371/journal.pone.0178334 PMC5444798 28542557

[39] Ramirez LA , Sullivan JC. Sex differences in hypertension: where we have been and where we are going. *Am J Hypertens* 2018;31:1247–54. 30299518 10.1093/ajh/hpy148 PMC6233684

[40] Ebert SN , Liu XK, Woosley RL. Female gender as a risk factor for drug-induced cardiac arrhythmias: evaluation of clinical and experimental evidence. *J Womens Health* 1998;7:547–57. 9650155 10.1089/jwh.1998.7.547

[41] Lam CSP , Arnott C, Beale AL et al. Sex differences in heart failure. *Eur Heart J* 2019;40:3859–3868c. 31800034 10.1093/eurheartj/ehz835

[42] Talbot CPJ , Dolinsky VW. Sex differences in the developmental origins of cardiometabolic disease following exposure to maternal obesity and gestational diabetes^1^. *Appl Physiol Nutr Metab* 2019;44:687–95. 30500266 10.1139/apnm-2018-0667

[43] Do Carmo JM , Omoto ACM, Dai X et al. Sex differences in the impact of parental obesity on offspring cardiac SIRT3 expression, mitochondrial efficiency, and diastolic function early in life. *Am J Physiol Heart Circ Physiol* 2021;321:H485–9. 34296964 10.1152/ajpheart.00176.2021 PMC8461840

[44] Gray C , Gardiner SM, Elmes M et al. Excess maternal salt or fructose intake programmes sex-specific, stress- and fructose-sensitive hypertension in the offspring. *Br J Nutr* 2016;115:594–604. 26653028 10.1017/S0007114515004936

[45] Obi IE , McPherson KC, Pollock JS. Childhood adversity and mechanistic links to hypertension risk in adulthood. *Br J Pharmacol* 2019;176:1932–50. 30656638 10.1111/bph.14576 PMC6534788

[46] Sol CM , Santos S, Asimakopoulos AG et al. Associations of maternal phthalate and bisphenol urine concentrations during pregnancy with childhood blood pressure in a population-based prospective cohort study. *Environ Int* 2020;138:105677. 10.1016/j.envint.2020.105677 PMC7354351 32220816

[47] Zhang A , Hu H, Sánchez BN et al. Association between prenatal lead exposure and blood pressure in children. *Environ Health Perspect* 2012;120:445–50. 21947582 10.1289/ehp.1103736 PMC3295346

[48] Ma S , Xu C, Ma J et al. Association between perfluoroalkyl substance concentrations and blood pressure in adolescents. *Environ Pollut* 2019;254:112971. 10.1016/j.envpol.2019.112971 31394346

[49] Reik W , Collick A, Norris ML et al. Genomic imprinting determines methylation of parental alleles in transgenic mice. *Nature* 1987;328:248–51. 3600805 10.1038/328248a0

[50] Jones PA , Taylor SM, Mohandas T et al. Cell cycle-specific reactivation of an inactive X-chromosome locus by 5-azadeoxycytidine. *Proc Natl Acad Sci USA* 1982;79:1215–9. 6175964 10.1073/pnas.79.4.1215 PMC345932

[51] Mohandas T , Sparkes RS, Shapiro LJ. Reactivation of an inactive human X chromosome: evidence for X inactivation by DNA methylation. *Science* 1981;211:393–6. 6164095 10.1126/science.6164095

[52] Yoder JA , Walsh CP, Bestor TH. Cytosine methylation and the ecology of intragenomic parasites. *Trends Genet* 1997;13:335–40. 9260521 10.1016/s0168-9525(97)01181-5

[53] Ball MP , Li JB, Gao Y et al. Targeted and genome-scale strategies reveal gene-body methylation signatures in human cells. *Nat Biotechnol* 2009;27:361–8. 19329998 10.1038/nbt.1533 PMC3566772

[54] Jones PA , Laird PW. Cancer epigenetics comes of age. *Nat Genet* 1999;21:163–7. 9988266 10.1038/5947

[55] Ito S , Shen L, Dai Q et al. Tet proteins can convert 5-methylcytosine to 5-formylcytosine and 5-carboxylcytosine. *Science* 2011;333:1300–3. 21778364 10.1126/science.1210597 PMC3495246

[56] Ito S , D’Alessio AC, Taranova OV et al. Role of Tet proteins in 5mC to 5hmC conversion, ES-cell self-renewal and inner cell mass specification. *Nature* 2010;466:1129–33. 20639862 10.1038/nature09303 PMC3491567

[57] Amouroux R , Nashun B, Shirane K et al. De novo DNA methylation drives 5hmC accumulation in mouse zygotes. *Nat Cell Biol* 2016;18:225–33. 26751286 10.1038/ncb3296 PMC4765106

[58] Globisch D , Münzel M, Müller M et al. Tissue distribution of 5-hydroxymethylcytosine and search for active demethylation intermediates. *PLOS One* 2010;5:e15367. 10.1371/journal.pone.0015367 PMC3009720 21203455

[59] Hahn MA , Szabó PE, Pfeifer GP. 5-Hydroxymethylcytosine: a stable or transient DNA modification? *Genomics* 2014;104:314–23. 25181633 10.1016/j.ygeno.2014.08.015 PMC4252803

[60] Wu H , D’Alessio AC, Ito S et al. Genome-wide analysis of 5-hydroxymethylcytosine distribution reveals its dual function in transcriptional regulation in mouse embryonic stem cells. *Genes Dev* 2011;25:679–84. 21460036 10.1101/gad.2036011 PMC3070931

[61] Kochmanski JJ , Marchlewicz EH, Cavalcante RG et al. Longitudinal effects of developmental bisphenol A exposure on epigenome-wide DNA hydroxymethylation at imprinted loci in mouse blood. *Environ Health Perspect* 2018;126:077006. 10.1289/EHP3441 PMC6108846 30044229

[62] Rygiel CA , Goodrich JM, Solano-González M et al. Prenatal lead (Pb) exposure and peripheral blood DNA methylation (5mC) and hydroxymethylation (5hmC) in Mexican adolescents from the ELEMENT birth cohort. *Environ Health Perspect* 2021;129:67002. 10.1289/EHP8507 PMC8216410 34152198

[63] Kafri T , Ariel M, Brandeis M et al. Developmental pattern of gene-specific DNA methylation in the mouse embryo and germ line. *Genes Dev* 1992;6:705–14. 1577268 10.1101/gad.6.5.705

[64] Monk M , Boubelik M, Lehnert S. Temporal and regional changes in DNA methylation in the embryonic, extraembryonic and germ cell lineages during mouse embryo development. *Development* 1987;99:371–82. 3653008 10.1242/dev.99.3.371

[65] Rougier N , Bourc’his D, Gomes DM et al. Chromosome methylation patterns during mammalian preimplantation development. *Genes Dev* 1998;12:2108–13. 9679055 10.1101/gad.12.14.2108 PMC317005

[66] Guo F , Li X, Liang D et al. Active and passive demethylation of male and female pronuclear DNA in the mammalian zygote. *Cell Stem Cell* 2014;15:447–59. 25220291 10.1016/j.stem.2014.08.003

[67] Seisenberger S , Andrews S, Krueger F et al. The dynamics of genome-wide DNA methylation reprogramming in mouse primordial germ cells. *Mol Cell* 2012;48:849–62. 23219530 10.1016/j.molcel.2012.11.001 PMC3533687

[68] Nilsson EE , Ben Maamar M, Skinner MK. Role of epigenetic transgenerational inheritance in generational toxicology. *Environ Epigenet* 2022;8:dvac001. 10.1093/eep/dvac001 PMC8848501 35186326

[69] Weigel D , Colot V. Epialleles in plant evolution. *Genome Biol* 2012;13:249. 10.1186/gb-2012-13-10-249 PMC3491404 23058244

[70] Li Z , Yu Z, Yin D. Multi- and trans-generational disturbances of perfluorobutane sulfonate and perfluorohexane sulfonate on lipid metabolism in *Caenorhabditis elegans*. *Chemosphere* 2021;280:130666. 10.1016/j.chemosphere.2021.130666 33945899

[71] Seong KH , Li D, Shimizu H et al. Inheritance of stress-induced, ATF-2-dependent epigenetic change. *Cell* 2011;145:1049–61. 21703449 10.1016/j.cell.2011.05.029

[72] Horsthemke B . A critical view on transgenerational epigenetic inheritance in humans. *Nat Commun* 2018;9:2973. 10.1038/s41467-018-05445-5 PMC6065375 30061690

[73] Martos SN , Tang WY, Wang Z. Elusive inheritance: transgenerational effects and epigenetic inheritance in human environmental disease. *Prog Biophys Mol Biol* 2015;118:44–54. 25792089 10.1016/j.pbiomolbio.2015.02.011 PMC4784256

[74] Ponzio BF , Carvalho MHC, Fortes ZB et al. Implications of maternal nutrient restriction in transgenerational programming of hypertension and endothelial dysfunction across F1-F3 offspring. *Life Sci* 2012;90:571–7. 22365957 10.1016/j.lfs.2012.01.017

[75] Lombo M et al. Transgenerational inheritance of heart disorders caused by paternal bisphenol A exposure. *Environ Pollut* 2015;206:667–78. 26322593 10.1016/j.envpol.2015.08.016

[76] Kaati G , Bygren LO, Edvinsson S. Cardiovascular and diabetes mortality determined by nutrition during parents’ and grandparents’ slow growth period. *Eur J Hum Genet* 2002;10:682–8. 12404098 10.1038/sj.ejhg.5200859

[77] Bygren LO , Tinghög P, Carstensen J et al. Change in paternal grandmothers’ early food supply influenced cardiovascular mortality of the female grandchildren. *BMC Genet* 2014;15:12. 10.1186/1471-2156-15-12 PMC3929550 24552514

[78] Li J , Liu S, Li S et al. Prenatal exposure to famine and the development of hyperglycemia and type 2 diabetes in adulthood across consecutive generations: a population-based cohort study of families in Suihua. *China Am J Clin Nutr* 2017;105:221–7. 27927634 10.3945/ajcn.116.138792

[79] Serpeloni F , Radtke K, de Assis SG et al. Grandmaternal stress during pregnancy and DNA methylation of the third generation: an epigenome-wide association study. *Transl Psychiatry* 2017;7:e1202. 10.1038/tp.2017.153 PMC5611722 28809857

[80] Perroud N , Rutembesa E, Paoloni-Giacobino A et al. The Tutsi genocide and transgenerational transmission of maternal stress: epigenetics and biology of the HPA axis. *World J Biol Psychiatry* 2014;15:334–45. 24690014 10.3109/15622975.2013.866693

[81] Wolstenholme JT , Goldsby JA, Rissman EF. Transgenerational effects of prenatal bisphenol A on social recognition. *Horm Behav* 2013;64:833–9. 24100195 10.1016/j.yhbeh.2013.09.007 PMC3955720

[82] Manikkam M , Tracey R, Guerrero-Bosagna C et al. Plastics derived endocrine disruptors (BPA, DEHP and DBP) induce epigenetic transgenerational inheritance of obesity, reproductive disease and sperm epimutations. *PLOS One* 2013;8:e55387. 10.1371/journal.pone.0055387 PMC3554682 23359474

[83] Manikkam M , Haque MM, Guerrero-Bosagna C et al. Pesticide methoxychlor promotes the epigenetic transgenerational inheritance of adult-onset disease through the female germline. *PLOS One* 2014;9:e102091. 10.1371/journal.pone.0102091 PMC4109920 25057798

[84] Manikkam M , Guerrero-Bosagna C, Tracey R et al. Transgenerational actions of environmental compounds on reproductive disease and identification of epigenetic biomarkers of ancestral exposures. *PLOS One* 2012;7:e31901. 10.1371/journal.pone.0031901 PMC3289630 22389676

[85] Guerrero-Bosagna C , Covert TR, Haque MM et al. Epigenetic transgenerational inheritance of vinclozolin induced mouse adult onset disease and associated sperm epigenome biomarkers. *Reprod Toxicol* 2012;34:694–707. 23041264 10.1016/j.reprotox.2012.09.005 PMC3513496

[86] Tracey R , Manikkam M, Guerrero-Bosagna C et al. Hydrocarbons (jet fuel JP-8) induce epigenetic transgenerational inheritance of obesity, reproductive disease and sperm epimutations. *Reprod Toxicol* 2013;36:104–16. 23453003 10.1016/j.reprotox.2012.11.011 PMC3587983

[87] Kubsad D , Nilsson EE, King SE et al. Assessment of glyphosate induced epigenetic transgenerational inheritance of pathologies and sperm epimutations: generational toxicology. *Sci Rep* 2019;9:6372. 10.1038/s41598-019-42860-0 PMC6476885 31011160

[88] Chamorro-Garcia R , Sahu M, Abbey RJ et al. Transgenerational inheritance of increased fat depot size, stem cell reprogramming, and hepatic steatosis elicited by prenatal exposure to the obesogen tributyltin in mice. *Environ Health Perspect* 2013;121:359–66. 23322813 10.1289/ehp.1205701 PMC3621201

[89] Dunn GA , Bale TL. Maternal high-fat diet effects on third-generation female body size via the paternal lineage. *Endocrinology* 2011;152:2228–36. 21447631 10.1210/en.2010-1461 PMC3100614

[90] Apostolou A , Garcia-Esquinas E, Fadrowski JJ et al. Secondhand tobacco smoke: a source of lead exposure in US children and adolescents. *Am J Public Health* 2012;102:714–22. 21852639 10.2105/AJPH.2011.300161 PMC3489360

[91] Abadin H et al. *Toxicological Profile for Lead* . Atlanta: Agency for Toxic Substance and Disease Registry (US), 2007. 24049859

[92] Riaz MA , Nisa ZU, Anjum MS et al. Assessment of metals induced histopathological and gene expression changes in different organs of non-diabetic and diabetic rats. *Sci Rep* 2020;10:5897. 10.1038/s41598-020-62807-0 PMC7125094 32246071

[93] Ansari MA , Maayah ZH, Bakheet SA et al. The role of aryl hydrocarbon receptor signaling pathway in cardiotoxicity of acute lead intoxication in vivo and in vitro rat model. *Toxicology* 2013;306:40–9. 23391631 10.1016/j.tox.2013.01.024

[94] Davuljigari CB , Gottipolu RR. Late-life cardiac injury in rats following early life exposure to lead: reversal effect of nutrient metal mixture. *Cardiovasc Toxicol* 2020;20:249–60. 31541351 10.1007/s12012-019-09549-2

[95] Simoes MR , Preti SC, Azevedo BF et al. Low-level chronic lead exposure impairs neural control of blood pressure and heart rate in rats. *Cardiovasc Toxicol* 2017;17:190–9. 27272938 10.1007/s12012-016-9374-y

[96] Shvachiy L , Geraldes V, Amaro-Leal Â et al. Persistent effects on cardiorespiratory and nervous systems induced by long-term lead exposure: results from a longitudinal study. *Neurotox Res* 2020;37:857–70. 31997153 10.1007/s12640-020-00162-8

[97] Gaspar AF , Cordellini S. Combination therapy for the cardiovascular effects of perinatal lead exposure in young and adult rats. *Arq Bras Cardiol* 2014;103:219–30. 25076181 10.5935/abc.20140103 PMC4193069

[98] Sun Y , Zhang H, Xing X et al. Lead promotes abnormal angiogenesis induced by CCM3 gene defects via mitochondrial pathway. *J Dev Orig Health Dis* 2018;9:182–90. 29110746 10.1017/S2040174417000782

[99] Chen Z et al. Lead (Pb) exposure and heart failure risk. *Environ Sci Pollut Res Int* 2021;28:28833–47. 33840028 10.1007/s11356-021-13725-9

[100] Faulk C , Barks A, Sánchez BN et al. Perinatal lead (Pb) exposure results in sex-specific effects on food intake, fat, weight, and insulin response across the murine life-course. *PLOS One* 2014;9:e104273. 10.1371/journal.pone.0104273 PMC4126699 25105421

[101] Dearth RK , Hiney JK, Srivastava V et al. Effects of lead (Pb) exposure during gestation and lactation on female pubertal development in the rat. *Reprod Toxicol* 2002;16:343–52. 12220594 10.1016/s0890-6238(02)00037-0

[102] Corpas I , Benito MJ, Marquina D et al. Gestational and lactational lead intoxication produces alterations in the hepatic system of rat pups. *Ecotoxicol Environ Saf* 2002;51:35–43. 11800548 10.1006/eesa.2001.2102

[103] Aladaileh SH , Khafaga AF, Abd El-Hack ME et al. Spirulina platensis ameliorates the sub chronic toxicities of lead in rabbits via anti-oxidative, anti- inflammatory, and immune stimulatory properties. *Sci Total Environ* 2020;701:134879. 10.1016/j.scitotenv.2019.134879 31734488

[104] Xu LH , Mu-F-F, Zhao J-H et al. Lead induces apoptosis and histone hyperacetylation in rat cardiovascular tissues. *PLOS One* 2015;10:e0129091. 10.1371/journal.pone.0129091 PMC4468051 26075388

[105] Carmignani M , Boscolo P, Poma A et al. Kininergic system and arterial hypertension following chronic exposure to inorganic lead. *Immunopharmacology* 1999;44:105–10. 10604532 10.1016/s0162-3109(99)00115-0

[106] Menke A , Muntner P, Batuman V et al. Blood lead below 0.48 micromol/L (10 microg/dL) and mortality among US adults. *Circulation* 2006;114:1388–94. 16982939 10.1161/CIRCULATIONAHA.106.628321

[107] Navas-Acien A , Guallar E, Silbergeld EK et al. Lead exposure and cardiovascular disease—a systematic review. *Environ Health Perspect* 2007;115:472–82. 17431501 10.1289/ehp.9785 PMC1849948

[108] Kieltucki J , Dobrakowski M, Pawlas N et al. The analysis of QT interval and repolarization morphology of the heart in chronic exposure to lead. *Hum Exp Toxicol* 2017;36:1081–6. 27903879 10.1177/0960327116680277

[109] Chen Z , Huo X, Zhang S et al. Relations of blood lead levels to echocardiographic left ventricular structure and function in preschool children. *Chemosphere* 2021;268:128793. 10.1016/j.chemosphere.2020.128793 33143894

[110] Liu Z , Yu Y, Li X et al. Maternal lead exposure and risk of congenital heart defects occurrence in offspring. *Reprod Toxicol* 2015;51:1–6. 25462788 10.1016/j.reprotox.2014.11.002

[111] Al-Sabbak M , Sadik Ali S, Savabi O et al. Metal contamination and the epidemic of congenital birth defects in Iraqi cities. *Bull Environ Contam Toxicol* 2012;89:937–44. 22983726 10.1007/s00128-012-0817-2 PMC3464374

[112] Ou Y , Bloom MS, Nie Z et al. Associations between toxic and essential trace elements in maternal blood and fetal congenital heart defects. *Environ Int* 2017;106:127–34. 28645012 10.1016/j.envint.2017.05.017

[113] Kundak AA , Pektas A, Zenciroglu A et al. Do toxic metals and trace elements have a role in the pathogenesis of conotruncal heart malformations? *Cardiol Young* 2017;27:312–7. 27751200 10.1017/S1047951116000536

[114] Salehi F , Darmiani K, Nakhaee S et al. Comparison of blood lead concentrations in mothers of children with congenital heart disease and mothers of healthy children. *Biol Trace Elem Res* 2022;200:2001–7. 10.1007/s12011-021-02813-z 34231195

[115] Gump BB , Stewart P, Reihman J et al. Prenatal and early childhood blood lead levels and cardiovascular functioning in 9 (1/2) year old children. *Neurotoxicol Teratol* 2005;27:655–65. 15919179 10.1016/j.ntt.2005.04.002

[116] Gump BB , MacKenzie JA, Bendinskas K et al. Low-level Pb and cardiovascular responses to acute stress in children: the role of cardiac autonomic regulation. *Neurotoxicol Teratol* 2011;33:212–9. 20934510 10.1016/j.ntt.2010.10.001 PMC3030645

[117] Sanders AP , Svensson K, Gennings C et al. Prenatal lead exposure modifies the effect of shorter gestation on increased blood pressure in children. *Environ Int* 2018;120:464–71. 30145310 10.1016/j.envint.2018.08.038 PMC6354251

[118] Howe CG , Margetaki K, Vafeiadi M et al. Prenatal metal mixtures and child blood pressure in the Rhea mother-child cohort in Greece. *Environ Health* 2021;20:1. 10.1186/s12940-020-00685-9 PMC7789252 33407552

[119] Cassidy-Bushrow AE , Sitarik AR, Havstad S et al. Burden of higher lead exposure in African-Americans starts in utero and persists into childhood. *Environ Int* 2017;108:221–7. 28886415 10.1016/j.envint.2017.08.021 PMC5623116

[120] Skroder H , Hawkesworth S, Moore SE et al. Prenatal lead exposure and childhood blood pressure and kidney function. *Environ Res* 2016;151:628–34. 27611993 10.1016/j.envres.2016.08.028

[121] Liu Y , Ettinger AS, Téllez-Rojo M et al. Prenatal lead exposure, type 2 diabetes, and cardiometabolic risk factors in Mexican children at age 10-18 years. *J Clin Endocrinol Metab* 2020;105:210–18. 10.1210/clinem/dgz038 PMC7037075 31608940

[122] Pirkle JL et al. The decline in blood lead levels in the United States. The National Health and Nutrition Examination Surveys (NHANES). *JAMA* 1994;272:284–91. 8028141

[123] Bollati V , Marinelli B, Apostoli P et al. Exposure to metal-rich particulate matter modifies the expression of candidate microRNAs in peripheral blood leukocytes. *Environ Health Perspect* 2010;118:763–8. 20061215 10.1289/ehp.0901300 PMC2898851

[124] Montrose L , Goodrich JM, Morishita M et al. Neonatal lead (Pb) exposure and DNA methylation profiles in dried bloodspots. *Int J Environ Res Public Health* 2020;17:6775. 10.3390/ijerph17186775 PMC7559513 32957503

[125] Sen A , Cingolani P, Senut M-C et al. Lead exposure induces changes in 5-hydroxymethylcytosine clusters in CpG islands in human embryonic stem cells and umbilical cord blood. *Epigenetics* 2015;10:607–21. 26046694 10.1080/15592294.2015.1050172 PMC4623482

[126] Sen A , Heredia N, Senut M-C et al. Early life lead exposure causes gender-specific changes in the DNA methylation profile of DNA extracted from dried blood spots. *Epigenomics* 2015;7:379–93. 26077427 10.2217/epi.15.2 PMC4501025

[127] Goodrich JM , Sánchez BN, Dolinoy DC et al. Quality control and statistical modeling for environmental epigenetics: a study on in utero lead exposure and DNA methylation at birth. *Epigenetics* 2015;10:19–30. 25580720 10.4161/15592294.2014.989077 PMC4622744

[128] Sen A , Heredia N, Senut M-C et al. Multigenerational epigenetic inheritance in humans: DNA methylation changes associated with maternal exposure to lead can be transmitted to the grandchildren. *Sci Rep* 2015;5:14466. 10.1038/srep14466 PMC4586440 26417717

[129] Engstrom K , Rydbeck F, Kippler M et al. Prenatal lead exposure is associated with decreased cord blood DNA methylation of the glycoprotein VI gene involved in platelet activation and thrombus formation. *Environ Epigenet* 2015;1:dvv007. 10.1093/eep/dvv007 PMC5804686 29492281

[130] MacGrogan D , Munch J, de la Pompa JL. Notch and interacting signalling pathways in cardiac development, disease, and regeneration. *Nat Rev Cardiol* 2018;15:685–704. 30287945 10.1038/s41569-018-0100-2

[131] Wieneke H , Svendsen JH, Lande J et al. Polymorphisms in the GNAS gene as predictors of ventricular tachyarrhythmias and sudden cardiac death: results from the DISCOVERY trial and Oregon sudden unexpected death study. *J Am Heart Assoc* 2016;5:e003905. 10.1161/JAHA.116.003905 PMC5210425 27895044

[132] Wu S , Hivert M-F, Cardenas A et al. Exposure to low levels of lead in utero and umbilical cord blood DNA methylation in project viva: an epigenome-wide association study. *Environ Health Perspect* 2017;125:087019. 10.1289/EHP1246 PMC5783669 28858830

[133] Nye MD , King KE, Darrah TH et al. Maternal blood lead concentrations, DNA methylation of MEG3 DMR regulating the DLK1/MEG3 imprinted domain and early growth in a multiethnic cohort. *Environ Epigenet* 2016;2:dvv009. 10.1093/eep/dvv009 PMC5258134 28123784

[134] Piccoli MT , Gupta SK, Viereck J et al. Inhibition of the cardiac fibroblast-enriched lncRNA Meg3 prevents cardiac fibrosis and diastolic dysfunction. *Circ Res* 2017;121:575–83. 28630135 10.1161/CIRCRESAHA.117.310624

[135] He C , Yang W, Yang J et al. Long noncoding RNA MEG3 negatively regulates proliferation and angiogenesis in vascular endothelial cells. *DNA Cell Biol* 2017;36:475–81. 28418724 10.1089/dna.2017.3682

[136] Li Y , Xie C, Murphy SK et al. Lead exposure during early human development and DNA methylation of imprinted gene regulatory elements in adulthood. *Environ Health Perspect* 2016;124:666–73. 26115033 10.1289/ehp.1408577 PMC4858407

[137] Rodriguez S et al. Haplotypic analyses of the IGF2-INS-TH gene cluster in relation to cardiovascular risk traits. *Hum Mol Genet* 2004;13:715–25. 14749349 10.1093/hmg/ddh070

[138] Sanchez OF , Lee J, Yu King Hing N et al. Lead (Pb) exposure reduces global DNA methylation level by non-competitive inhibition and alteration of dnmt expression. *Metallomics* 2017;9:149–60. 27934997 10.1039/c6mt00198j

[139] Park K , Han EJ, Ahn G et al. Effects of thermal stress-induced lead (Pb) toxicity on apoptotic cell death, inflammatory response, oxidative defense, and DNA methylation in zebrafish (*Danio rerio*) embryos. *Aquat Toxicol* 2020;224:105479. 10.1016/j.aquatox.2020.105479 32417751

[140] Dunn J , Simmons R, Thabet S et al. The role of epigenetics in the endothelial cell shear stress response and atherosclerosis. *Int J Biochem Cell Biol* 2015;67:167–76. 25979369 10.1016/j.biocel.2015.05.001 PMC4592147

[141] Vujic A , Robinson EL, Ito M et al. Experimental heart failure modelled by the cardiomyocyte-specific loss of an epigenome modifier, DNMT3B. *J Mol Cell Cardiol* 2015;82:174–83. 25784084 10.1016/j.yjmcc.2015.03.007

[142] Peng C , Deng Q, Li Z et al. Risk-association of DNMT1 gene polymorphisms with coronary artery disease in Chinese Han population. *Int J Mol Sci* 2014;15:22694–705. 25493477 10.3390/ijms151222694 PMC4284731

[143] Montrose L , Faulk C, Francis J et al. Perinatal lead (Pb) exposure results in sex and tissue-dependent adult DNA methylation alterations in murine IAP transposons. *Environ Mol Mutagen* 2017;58:540–50. 28833526 10.1002/em.22119 PMC5784428

[144] Dou JF , Farooqui Z, Faulk CD et al. Perinatal lead (Pb) exposure and cortical neuron-specific DNA methylation in male mice. *Genes (Basel)* 2019;10:274. 10.3390/genes10040274 PMC6523909 30987383

[145] Schneider JS , Kidd SK, Anderson DW. Influence of developmental lead exposure on expression of DNA methyltransferases and methyl cytosine-binding proteins in hippocampus. *Toxicol Lett* 2013;217:75–81. 23246732 10.1016/j.toxlet.2012.12.004 PMC3545007

[146] Svoboda LK , Wang K, Jones TR et al. Sex-specific alterations in cardiac DNA methylation in adult mice by perinatal lead exposure. *Int J Environ Res Public Health* 2021;18:577. 10.3390/ijerph18020577 PMC7826866 33445541

[147] Sunderland EM , Hu XC, Dassuncao C et al. A review of the pathways of human exposure to poly- and perfluoroalkyl substances (PFASs) and present understanding of health effects. *J Expo Sci Environ Epidemiol* 2019;29:131–47. 30470793 10.1038/s41370-018-0094-1 PMC6380916

[148] Lv N , Yuan J, Ji A et al. Perfluorooctanoic acid-induced toxicities in chicken embryo primary cardiomyocytes: roles of PPAR alpha and Wnt5a/Frizzled2. *Toxicol Appl Pharmacol* 2019;381:114716. 10.1016/j.taap.2019.114716 31445018

[149] Jiang Q , Lust RM, Strynar MJ et al. Perflurooctanoic acid induces developmental cardiotoxicity in chicken embryos and hatchlings. *Toxicology* 2012;293:97–106. 22273728 10.1016/j.tox.2012.01.005

[150] Xu X , Ni H, Guo Y et al. Hexafluoropropylene oxide dimer acid (HFPO-DA) induced developmental cardiotoxicity and hepatotoxicity in hatchling chickens: roles of peroxisome proliferator activated receptor alpha. *Environ Pollut* 2021;290:118112. 10.1016/j.envpol.2021.118112 34500398

[151] Kim M , Son J, Park MS et al. In vivo evaluation and comparison of developmental toxicity and teratogenicity of perfluoroalkyl compounds using Xenopus embryos. *Chemosphere* 2013;93:1153–60. 23910242 10.1016/j.chemosphere.2013.06.053

[152] Zeng HC , He Q-Z, Li-Y-Y et al. Prenatal exposure to PFOS caused mitochondia-mediated apoptosis in heart of weaned rat. *Environ Toxicol* 2015;30:1082–90. 24616003 10.1002/tox.21981

[153] Salimi A , Nikoosiar Jahromi M, Pourahmad J. Maternal exposure causes mitochondrial dysfunction in brain, liver, and heart of mouse fetus: an explanation for perfluorooctanoic acid induced abortion and developmental toxicity. *Environ Toxicol* 2019;34:878–85. 31037826 10.1002/tox.22760

[154] Yu J , Cheng W, Jia M et al. Toxicity of perfluorooctanoic acid on zebrafish early embryonic development determined by single-cell RNA sequencing. *J Hazard Mater* 2021;427:127888. 10.1016/j.jhazmat.2021.127888 34862108

[155] Conley JM , Lambright CS, Evans N et al. Hexafluoropropylene oxide-dimer acid (HFPO-DA or GenX) alters maternal and fetal glucose and lipid metabolism and produces neonatal mortality, low birthweight, and hepatomegaly in the Sprague-Dawley rat. *Environ Int* 2021;146:106204. 10.1016/j.envint.2020.106204 PMC7775906 33126064

[156] Hines EP , White SS, Stanko JP et al. Phenotypic dichotomy following developmental exposure to perfluorooctanoic acid (PFOA) in female CD-1 mice: low doses induce elevated serum leptin and insulin, and overweight in mid-life. *Mol Cell Endocrinol* 2009;304:97–105. 19433254 10.1016/j.mce.2009.02.021

[157] Gardener H , Sun Q, Grandjean P. PFAS concentration during pregnancy in relation to cardiometabolic health and birth outcomes. *Environ Res* 2021;192:110287. 10.1016/j.envres.2020.110287 PMC7736328 33038367

[158] Yu G , Jin M, Huang Y et al. Environmental exposure to perfluoroalkyl substances in early pregnancy, maternal glucose homeostasis and the risk of gestational diabetes: a prospective cohort study. *Environ Int* 2021;156:106621. 10.1016/j.envint.2021.106621 33984575

[159] Birukov A , Andersen LB, Andersen MS et al. Exposure to perfluoroalkyl substances and blood pressure in pregnancy among 1436 women from the Odense Child Cohort. *Environ Int* 2021;151:106442. 10.1016/j.envint.2021.106442 PMC11149831 33610053

[160] Borghese MM , Walker M, Helewa ME et al. Association of perfluoroalkyl substances with gestational hypertension and preeclampsia in the MIREC study. *Environ Int* 2020;141:105789. 10.1016/j.envint.2020.105789 32408216

[161] Christensen JJ , Retterstøl K, Godang K et al. LDL cholesterol in early pregnancy and offspring cardiovascular disease risk factors. *J Clin Lipidol* 2016;10:1369–78. 27919354 10.1016/j.jacl.2016.08.016

[162] Perak AM , Lancki N, Kuang A et al. Associations of maternal cardiovascular health in pregnancy with offspring cardiovascular health in early adolescence. *JAMA* 2021;325:658–68. 33591345 10.1001/jama.2021.0247 PMC7887661

[163] Yu Y , Arah OA, Liew Z et al. Maternal diabetes during pregnancy and early onset of cardiovascular disease in offspring: population based cohort study with 40 years of follow-up. *BMJ* 2019;367:l6398. 10.1136/bmj.l6398 PMC6891797 31801789

[164] Stojanovska V , Scherjon SA, Plosch T. Preeclampsia as modulator of offspring health. *Biol Reprod* 2016;94:53. 10.1095/biolreprod.115.135780 26792940

[165] Ou Y , Zeng X, Lin S et al. Gestational exposure to perfluoroalkyl substances and congenital heart defects: a nested case-control pilot study. *Environ Int* 2021;154:106567. 10.1016/j.envint.2021.106567 33882431

[166] Savitz DA , Stein CR, Bartell SM et al. Perfluorooctanoic acid exposure and pregnancy outcome in a highly exposed community. *Epidemiology* 2012;23:386–92. 22370857 10.1097/EDE.0b013e31824cb93b PMC3321117

[167] Stein CR , Savitz DA, Elston B et al. Perfluorooctanoate exposure and major birth defects. *Reprod Toxicol* 2014;47:15–20. 24803403 10.1016/j.reprotox.2014.04.006 PMC4117925

[168] Nolan LA , Nolan JM, Shofer FS et al. Congenital anomalies, labor/delivery complications, maternal risk factors and their relationship with perfluorooctanoic acid (PFOA)-contaminated public drinking water. *Reprod Toxicol* 2010;29:147–55. 19897029 10.1016/j.reprotox.2009.10.012 PMC3038105

[169] Khalil N , Ebert JR, Honda M et al. Perfluoroalkyl substances, bone density, and cardio-metabolic risk factors in obese 8-12 year old children: a pilot study. *Environ Res* 2018;160:314–21. 29040951 10.1016/j.envres.2017.10.014

[170] Averina M , Brox J, Huber S et al. Exposure to perfluoroalkyl substances (PFAS) and dyslipidemia, hypertension and obesity in adolescents. The Fit Futures study. *Environ Res* 2021;195:110740. 10.1016/j.envres.2021.110740 33460636

[171] Manzano-Salgado CB , Casas M, Lopez-Espinosa M-J et al. Prenatal exposure to perfluoroalkyl substances and cardiometabolic risk in children from the Spanish INMA birth cohort study. *Environ Health Perspect* 2017;125:097018. 10.1289/EHP1330 PMC5915205 28934720

[172] Canova C , Di Nisio A, Barbieri G et al. PFAS concentrations and cardiometabolic traits in highly exposed children and adolescents. *Int J Environ Res Public Health* 2021;18:12881. 10.3390/ijerph182412881 PMC8701234 34948492

[173] Spratlen MJ , Perera FP, Lederman SA et al. The association between perfluoroalkyl substances and lipids in cord blood. *J Clin Endocrinol Metab* 2020;105:43–54. 10.1210/clinem/dgz024 PMC6936966 31536623

[174] Alderete TL , Jin R, Walker DI et al. Perfluoroalkyl substances, metabolomic profiling, and alterations in glucose homeostasis among overweight and obese Hispanic children: a proof-of-concept analysis. *Environ Int* 2019;126:445–53. 30844580 10.1016/j.envint.2019.02.047 PMC6555482

[175] Mora AM , Fleisch AF, Rifas-Shiman SL et al. Early life exposure to per- and polyfluoroalkyl substances and mid-childhood lipid and alanine aminotransferase levels. *Environ Int* 2018;111:1–13. 29156323 10.1016/j.envint.2017.11.008 PMC5801004

[176] Domazet SL , Jensen TK, Wedderkopp N et al. Exposure to perfluoroalkylated substances (PFAS) in relation to fitness, physical activity, and adipokine levels in childhood: the European Youth Heart Study. *Environ Res* 2020;191:110110. 10.1016/j.envres.2020.110110 32871146

[177] Halldorsson TI , Rytter D, Haug LS et al. Prenatal exposure to perfluorooctanoate and risk of overweight at 20 years of age: a prospective cohort study. *Environ Health Perspect* 2012;120:668–73. 22306490 10.1289/ehp.1104034 PMC3346773

[178] Hoyer BB , Ramlau-Hansen CH, Vrijheid M et al. Anthropometry in 5- to 9-year-old Greenlandic and Ukrainian children in relation to prenatal exposure to perfluorinated alkyl substances. *Environ Health Perspect* 2015;123:841–6. 25809098 10.1289/ehp.1408881 PMC4529015

[179] Yeung EH , Bell EM, Sundaram R et al. Examining endocrine disruptors measured in newborn dried blood spots and early childhood growth in a prospective cohort. *Obesity (Silver Spring)* 2019;27:145–51. 30569634 10.1002/oby.22332 PMC6309795

[180] Andersen CS , Fei C, Gamborg M et al. Prenatal exposures to perfluorinated chemicals and anthropometric measures in infancy. *Am J Epidemiol* 2010;172:1230–7. 20940176 10.1093/aje/kwq289

[181] Wang Y , Adgent M, Su P-H et al. Prenatal exposure to perfluorocarboxylic acids (PFCAs) and fetal and postnatal growth in the Taiwan Maternal and Infant Cohort Study. *Environ Health Perspect* 2016;124:1794–800. 26895313 10.1289/ehp.1509998 PMC5089898

[182] Andersen CS , Fei C, Gamborg M et al. Prenatal exposures to perfluorinated chemicals and anthropometry at 7 years of age. *Am J Epidemiol* 2013;178:921–7. 23825166 10.1093/aje/kwt057

[183] Maisonet M , Terrell ML, McGeehin MA et al. Maternal concentrations of polyfluoroalkyl compounds during pregnancy and fetal and postnatal growth in British girls. *Environ Health Perspect* 2012;120:1432–7. 22935244 10.1289/ehp.1003096 PMC3491920

[184] Watkins DJ , Wellenius GA, Butler RA et al. Associations between serum perfluoroalkyl acids and LINE-1 DNA methylation. *Environ Int* 2014;63:71–6. 24263140 10.1016/j.envint.2013.10.018 PMC4181536

[185] Xu Y , Jurkovic-Mlakar S, Li Y et al. Association between serum concentrations of perfluoroalkyl substances (PFAS) and expression of serum microRNAs in a cohort highly exposed to PFAS from drinking water. *Environ Int* 2020;136:105446. 10.1016/j.envint.2019.105446 31926437

[186] Xu Y , Jurkovic-Mlakar S, Lindh CH et al. Associations between serum concentrations of perfluoroalkyl substances and DNA methylation in women exposed through drinking water: a pilot study in Ronneby, Sweden. *Environ Int* 2020;145:106148. 10.1016/j.envint.2020.106148 33007577

[187] Miura R , Araki A, Miyashita C et al. An epigenome-wide study of cord blood DNA methylations in relation to prenatal perfluoroalkyl substance exposure: the Hokkaido study. *Environ Int* 2018;115:21–8. 29544137 10.1016/j.envint.2018.03.004

[188] Ramirez Flores RO , Lanzer JD, Holland CH et al. Consensus transcriptional landscape of human end-stage heart failure. *J Am Heart Assoc* 2021;10:e019667. 10.1161/JAHA.120.019667 PMC8174362 33787284

[189] Toma M , Mak GJ, Chen V et al. Differentiating heart failure phenotypes using sex-specific transcriptomic and proteomic biomarker panels. *ESC Heart Fail* 2017;4:301–11. 28772032 10.1002/ehf2.12136 PMC5542716

[190] Claro V , Ferro A. Netrin-1: focus on its role in cardiovascular physiology and atherosclerosis. *JRSM Cardiovasc Dis* 2020;9:2048004020959574. 10.1177/2048004020959574 PMC7691900 33282228

[191] Shamis Y , Cullen DE, Liu L et al. Maternal and zygotic Zfp57 modulate NOTCH signaling in cardiac development. *Proc Natl Acad Sci U S A* 2015;112:E2020–9. 25848000 10.1073/pnas.1415541112 PMC4413352

[192] Leung YK , Ouyang B, Niu L et al. Identification of sex-specific DNA methylation changes driven by specific chemicals in cord blood in a Faroese birth cohort. *Epigenetics* 2018;13:290–300. 29560787 10.1080/15592294.2018.1445901 PMC5997167

[193] Guerrero-Preston R , Goldman LR, Brebi-Mieville P et al. Global DNA hypomethylation is associated with in utero exposure to cotinine and perfluorinated alkyl compounds. *Epigenetics* 2010;5:539–46. 20523118 10.4161/epi.5.6.12378 PMC3322495

[194] Liu CY et al. Prenatal perfluorooctyl sulfonate exposure and Alu DNA Hypomethylation in cord blood. *Int J Environ Res Public Health* 2018;15:1066. 10.3390/ijerph15061066 PMC6025582 29795014

[195] Robinson SL , Zeng X, Guan W et al. Perfluorooctanoic acid (PFOA) or perfluorooctane sulfonate (PFOS) and DNA methylation in newborn dried blood spots in the Upstate KIDS cohort. *Environ Res* 2021;194:110668. 10.1016/j.envres.2020.110668 PMC7946760 33387539

[196] Sonkar R , Kay MK, Choudhury M. PFOS modulates interactive epigenetic regulation in first-trimester human trophoblast cell line HTR-8/SVneo. *Chem Res Toxicol* 2019;32:2016–27. 31508952 10.1021/acs.chemrestox.9b00198

[197] van den Dungen MW , Murk AJ, Kok DE et al. Persistent organic pollutants alter DNA methylation during human adipocyte differentiation. *Toxicol In Vitro* 2017;40:79–87. 28011154 10.1016/j.tiv.2016.12.011

[198] Xu B , Ji X, Chen X et al. Effect of perfluorooctane sulfonate on pluripotency and differentiation factors in mouse embryoid bodies. *Toxicology* 2015;328:160–7. 25510869 10.1016/j.tox.2014.12.010

[199] Blanc M , Kärrman A, Kukucka P et al. Mixture-specific gene expression in zebrafish (*Danio rerio*) embryos exposed to perfluorooctane sulfonic acid (PFOS), perfluorohexanoic acid (PFHxA) and 3,3ʹ,4,4ʹ,5-pentachlorobiphenyl (PCB126). *Sci Total Environ* 2017;590-591:249–57. 28283292 10.1016/j.scitotenv.2017.02.232

[200] Heudorf U , Mersch-Sundermann V, Angerer J. Phthalates: toxicology and exposure. *Int J Hyg Environ Health* 2007;210:623–34. 17889607 10.1016/j.ijheh.2007.07.011

[201] Ghassabian A , Vandenberg L, Kannan K et al. Endocrine-disrupting chemicals and child health. *Annu Rev Pharmacol Toxicol* 2022;62:573–94. 34555290 10.1146/annurev-pharmtox-021921-093352

[202] Tang C , Luo C, Hua Y et al. Placental P-glycoprotein inhibition enhances susceptibility to di-(2-ethylhexyl)-phthalate induced cardiac malformations in mice: a possibly promising target for congenital heart defects prevention. *PLOS One* 2019;14:e0214873. 10.1371/journal.pone.0214873 PMC6516658 31086358

[203] Sun Y , Yang F, Liu Y et al. Di-2-ethylhexyl phthalate induces heart looping disorders during zebrafish development. *Toxicol Ind Health* 2021;37:391–7. 34047658 10.1177/07482337211019184

[204] Sun G , Liu K. Developmental toxicity and cardiac effects of butyl benzyl phthalate in zebrafish embryos. *Aquat Toxicol* 2017;192:165–70. 28961509 10.1016/j.aquatox.2017.09.020

[205] Sun G , Li Y. Exposure to DBP induces the toxicity in early development and adverse effects on cardiac development in zebrafish (*Danio rerio*). *Chemosphere* 2019;218:76–82. 30469006 10.1016/j.chemosphere.2018.11.095

[206] Mangala Priya V , Mayilvanan C, Akilavalli N et al. Lactational exposure of phthalate impairs insulin signaling in the cardiac muscle of F1 female albino rats. *Cardiovasc Toxicol* 2014;14:10–20. 24297258 10.1007/s12012-013-9233-z

[207] Parsanathan R , Maria Joseph A, Karundevi B. Postnatal exposure to di-(2-ethylhexyl)phthalate alters cardiac insulin signaling molecules and GLUT4^Ser488^ phosphorylation in male rat offspring. *J Cell Biochem* 2019;120:5802–12. 30362281 10.1002/jcb.27866

[208] Li W , Zhang W, Chang M et al. Quadrupole Orbitrap Mass Spectrometer-based metabonomic elucidation of influences of short-term di(2-ethylhexyl) phthalate exposure on cardiac metabolism in male mice. *Chem Res Toxicol* 2018;31:1185–94. 30284816 10.1021/acs.chemrestox.8b00184

[209] Posnack NG , Swift LM, Kay MW et al. Phthalate exposure changes the metabolic profile of cardiac muscle cells. *Environ Health Perspect* 2012;120:1243–51. 22672789 10.1289/ehp.1205056 PMC3440133

[210] Martinez-Arguelles DB , McIntosh M, Rohlicek CV et al. Maternal in utero exposure to the endocrine disruptor di-(2-ethylhexyl) phthalate affects the blood pressure of adult male offspring. *Toxicol Appl Pharmacol* 2013;266:95–100. 23142467 10.1016/j.taap.2012.10.027

[211] Kristensen DM , Skalkam ML, Audouze K et al. Many putative endocrine disruptors inhibit prostaglandin synthesis. *Environ Health Perspect* 2011;119:534–41. 21081300 10.1289/ehp.1002635 PMC3080937

[212] Chen CW , Tang S-Y, Hwang J-S et al. Association between levels of urine di-(2-ethylhexyl)phthalate metabolites and heart rate variability in young adults. *Toxics* 2021;9:351. 10.3390/toxics9120351 PMC8709404 34941785

[213] Su TC , Hwang-J-J, Sun C-W et al. Urinary phthalate metabolites, coronary heart disease, and atherothrombotic markers. *Ecotoxicol Environ Saf* 2019;173:37–44. 30753939 10.1016/j.ecoenv.2019.02.021

[214] Su TC , Hwang J-S, Torng P-L et al. Phthalate exposure increases subclinical atherosclerosis in young population. *Environ Pollut* 2019;250:586–93. 31026707 10.1016/j.envpol.2019.04.006

[215] Wang C , Zhan Y, Wang F et al. Parental occupational exposures to endocrine disruptors and the risk of simple isolated congenital heart defects. *Pediatr Cardiol* 2015;36:1024–37. 25628158 10.1007/s00246-015-1116-6

[216] Snijder CA , Vlot IJ, Burdorf A et al. Congenital heart defects and parental occupational exposure to chemicals. *Hum Reprod* 2012;27:1510–7. 22357765 10.1093/humrep/des043

[217] Sol CM , Santos S, Duijts L et al. Fetal exposure to phthalates and bisphenols and childhood general and organ fat. A population-based prospective cohort study. *Int J Obes (Lond)* 2020;44:2225–35. 32920592 10.1038/s41366-020-00672-7

[218] Yao Y , Chen D-Y, Yin J-W et al. Phthalate exposure linked to high blood pressure in Chinese children. *Environ Int* 2020;143:105958. 10.1016/j.envint.2020.105958 32688158

[219] Trasande L , Sathyanarayana S, Spanier AJ et al. Urinary phthalates are associated with higher blood pressure in childhood. *J Pediatr* 2013;163:747–53. 23706605 10.1016/j.jpeds.2013.03.072 PMC4074773

[220] Bowman A , Peterson KE, Dolinoy DC et al. Phthalate exposures, DNA methylation and adiposity in Mexican children through adolescence. *Front Public Health* 2019;7:162. 10.3389/fpubh.2019.00162 PMC6593088 31275917

[221] Tran V , Tindula G, Huen K et al. Prenatal phthalate exposure and 8-isoprostane among Mexican-American children with high prevalence of obesity. *J Dev Orig Health Dis* 2017;8:196–205. 28031075 10.1017/S2040174416000763 PMC5332297

[222] Lin CY , Lee H-L, Hwang Y-T et al. The association between urine di-(2-ethylhexyl) phthalate metabolites, global DNA methylation, and subclinical atherosclerosis in a young Taiwanese population. *Environ Pollut* 2020;265:114912. 10.1016/j.envpol.2020.114912 32540595

[223] Huen K , Calafat AM, Bradman A et al. Maternal phthalate exposure during pregnancy is associated with DNA methylation of LINE-1 and Alu repetitive elements in Mexican-American children. *Environ Res* 2016;148:55–62. 27019040 10.1016/j.envres.2016.03.025 PMC4874877

[224] Zhao Y , Shi H-J, Xie C-M et al. Prenatal phthalate exposure, infant growth, and global DNA methylation of human placenta. *Environ Mol Mutagen* 2015;56:286–92. 25327576 10.1002/em.21916

[225] Muka T , Koromani F, Portilla E et al. The role of epigenetic modifications in cardiovascular disease: a systematic review. *Int J Cardiol* 2016;212:174–83. 27038728 10.1016/j.ijcard.2016.03.062

[226] Barker DJ , Osmond C, Winter PD et al. Weight in infancy and death from ischaemic heart disease. *Lancet* 1989;2:577–80. 2570282 10.1016/s0140-6736(89)90710-1

[227] LaRocca J , Binder AM, McElrath TF et al. The impact of first trimester phthalate and phenol exposure on IGF2/H19 genomic imprinting and birth outcomes. *Environ Res* 2014;133:396–406. 24972507 10.1016/j.envres.2014.04.032 PMC4155603

[228] Zaina S , Pettersson L, Thomsen AB et al. Shortened life span, bradycardia, and hypotension in mice with targeted expression of an Igf2 transgene in smooth muscle cells. *Endocrinology* 2003;144:2695–703. 12746334 10.1210/en.2002-220944

[229] Omura J , Habbout K, Shimauchi T et al. Identification of long noncoding RNA H19 as a new biomarker and therapeutic target in right ventricular failure in pulmonary arterial hypertension. *Circulation* 2020;142:1464–84. 32698630 10.1161/CIRCULATIONAHA.120.047626

[230] Martinez-Ibarra A , Martínez-Razo LD, Vázquez-Martínez ER et al. Unhealthy levels of phthalates and bisphenol A in Mexican pregnant women with gestational diabetes and its association to altered expression of miRNAs involved with metabolic disease. *Int J Mol Sci* 2019;20:3343. 10.3390/ijms20133343 PMC6650872 31284700

[231] Schaedlich K , Schmidt J-S, Kwong WY et al. Impact of di-ethylhexylphthalate exposure on metabolic programming in P19 ECC-derived cardiomyocytes. *J Appl Toxicol* 2015;35:861–9. 25351189 10.1002/jat.3085

[232] Svoboda LK , Wang K, Cavalcante RG et al. Sex-specific programming of cardiac DNA methylation by developmental phthalate exposure. *Epigenet Insights* 2020;13:2516865720939971. 10.1177/2516865720939971 PMC7430087 32864567

[233] Day FR , Elks CE, Murray A et al. Puberty timing associated with diabetes, cardiovascular disease and also diverse health outcomes in men and women: the UK Biobank study. *Sci Rep* 2015;5:11208. 10.1038/srep11208 PMC4471670 26084728

[234] Rajesh P , Balasubramanian K. Phthalate exposure in utero causes epigenetic changes and impairs insulin signalling. *J Endocrinol* 2014;223:47–66. 25232145 10.1530/JOE-14-0111

[235] Mu X , Chen X, Liu J et al. A multi-omics approach reveals molecular mechanisms by which phthalates induce cardiac defects in zebrafish (*Danio rerio*). *Environ Pollut* 2020;265:113876. 10.1016/j.envpol.2019.113876 32806432

[236] Cheng H , Yan W. MiR-433 regulates myocardial ischemia reperfusion injury by targeting NDRG4 via the PI3K/Akt pathway. *Shock* 2020;54:802–9. 32187107 10.1097/SHK.0000000000001532

[237] Lin YC , Nunez V, Johns R et al. APOA5 gene polymorphisms and cardiovascular diseases: metaprediction in global populations. *Nurs Res* 2017;66:164–74. 28252576 10.1097/NNR.0000000000000207

[238] Kim DE , Noh S-M, Jeong S-W et al. NINJ2 SNP may affect the onset age of first-ever ischemic stroke without increasing silent cerebrovascular lesions. *BMC Res Notes* 2012;5:155. 10.1186/1756-0500-5-155 PMC3368733 22429733

[239] Robbins N , Koch SE, Rubinstein J. Targeting TRPV1 and TRPV2 for potential therapeutic interventions in cardiovascular disease. *Transl Res* 2013;161:469–76. 23453732 10.1016/j.trsl.2013.02.003

[240] Meyer DN , Crofts EJ, Akemann C et al. Developmental exposure to Pb^2+^ induces transgenerational changes to zebrafish brain transcriptome. *Chemosphere* 2020;244:125527. 10.1016/j.chemosphere.2019.125527 PMC7015790 31816550

[241] Sobolewski M , Abston K, Conrad K et al. Lineage- and sex-dependent behavioral and biochemical transgenerational consequences of developmental exposure to lead, prenatal stress, and combined lead and prenatal stress in mice. *Environ Health Perspect* 2020;128:27001. 10.1289/EHP4977 PMC7064322 32073883

[242] Manenschijn L , Schaap L, van Schoor NM et al. High long-term cortisol levels, measured in scalp hair, are associated with a history of cardiovascular disease. *J Clin Endocrinol Metab* 2013;98:2078–83. 23596141 10.1210/jc.2012-3663

[243] Chen L , Lam JCW, Hu C et al. Perfluorobutanesulfonate exposure skews sex ratio in fish and transgenerationally impairs reproduction. *Environ Sci Technol* 2019;53:8389–97. 31269390 10.1021/acs.est.9b01711

[244] Chowdhury MI , Sana T, Panneerselvan L et al. Acute toxicity and transgenerational effects of perfluorobutane sulfonate on *Caenorhabditis elegans*. *Environ Toxicol Chem* 2021;40:1973–82. 33792982 10.1002/etc.5055

[245] Shi G , Wang J, Guo H et al. Parental exposure to 6:2 chlorinated polyfluorinated ether sulfonate (F-53B) induced transgenerational thyroid hormone disruption in zebrafish. *Sci Total Environ* 2019;665:855–63. 30790758 10.1016/j.scitotenv.2019.02.198

[246] Lu Y , Wang J, Guo X et al. Perfluorooctanoic acid affects endocytosis involving clathrin light chain A and microRNA-133b-3p in mouse testes. *Toxicol Appl Pharmacol* 2017;318:41–8. 28126411 10.1016/j.taap.2017.01.014

[247] Leter G , Consales C, Eleuteri P et al. Exposure to perfluoroalkyl substances and sperm DNA global methylation in Arctic and European populations. *Environ Mol Mutagen* 2014;55:591–600. 24889506 10.1002/em.21874

[248] Yuan B , Wu W, Chen M et al. From the cover: metabolomics reveals a role of betaine in prenatal DBP exposure-induced epigenetic transgenerational failure of spermatogenesis in rats. *Toxicol Sci* 2017;158:356–66. 28898977 10.1093/toxsci/kfx092

[249] Doyle TJ , Bowman JL, Windell VL et al. Transgenerational effects of di-(2-ethylhexyl) phthalate on testicular germ cell associations and spermatogonial stem cells in mice. *Biol Reprod* 2013;88:112. 10.1095/biolreprod.112.106104 PMC4013901 23536373

[250] Rattan S , Brehm E, Gao L et al. Di(2-ethylhexyl) phthalate exposure during prenatal development causes adverse transgenerational effects on female fertility in mice. *Toxicol Sci* 2018;163:420–9. 29471507 10.1093/toxsci/kfy042 PMC5974785

[251] Meltzer D , Martinez–Arguelles DB, Campioli E et al. In utero exposure to the endocrine disruptor di(2-ethylhexyl) phthalate targets ovarian theca cells and steroidogenesis in the adult female rat. *Reprod Toxicol* 2015;51:47–56. 25530038 10.1016/j.reprotox.2014.12.005

[252] Brehm E , Zhou C, Gao L et al. Prenatal exposure to an environmentally relevant phthalate mixture accelerates biomarkers of reproductive aging in a multiple and transgenerational manner in female mice. *Reprod Toxicol* 2020;98:260–8. 33129917 10.1016/j.reprotox.2020.10.009 PMC7736276

[253] Honigberg MC , Zekavat SM, Aragam K et al. Association of premature natural and surgical menopause with incident cardiovascular disease. *JAMA* 2019;322:2411–21. 31738818 10.1001/jama.2019.19191 PMC7231649

[254] Quinnies KM , Doyle TJ, Kim KH et al. Transgenerational effects of di-(2-ethylhexyl) phthalate (DEHP) on stress hormones and behavior. *Endocrinology* 2015;156:3077–83. 26168342 10.1210/EN.2015-1326 PMC4541619

[255] International Consortium for Blood Pressure Genome-Wide Association, Studies et al. Genetic variants in novel pathways influence blood pressure and cardiovascular disease risk. *Nature* 2011;478:103–9. 21909115 10.1038/nature10405 PMC3340926

[256] Li L , Zhang T, Qin X-S et al. Exposure to diethylhexyl phthalate (DEHP) results in a heritable modification of imprint genes DNA methylation in mouse oocytes. *Mol Biol Rep* 2014;41:1227–35. 24390239 10.1007/s11033-013-2967-7

[257] Kloner RA , Carson C, Dobs A et al. Testosterone and cardiovascular disease. *J Am Coll Cardiol* 2016;67:545–57. 26846952 10.1016/j.jacc.2015.12.005

[258] Clayton JA , Collins FS. Policy: NIH to balance sex in cell and animal studies. *Nature* 2014;509:282–3. 24834516 10.1038/509282a PMC5101948

[259] Woitowich NC , Beery A, Woodruff T. A 10-year follow-up study of sex inclusion in the biological sciences. *Elife* 2020;9:e56344. 10.7554/eLife.56344 PMC7282816 32513386

